# Combined PD-L1/TGFβ blockade allows expansion and differentiation of stem cell-like CD8 T cells in immune excluded tumors

**DOI:** 10.1038/s41467-023-40398-4

**Published:** 2023-08-05

**Authors:** Alessandra Castiglioni, Yagai Yang, Katherine Williams, Alvin Gogineni, Ryan S. Lane, Amber W. Wang, Justin A. Shyer, Zhe Zhang, Stephanie Mittman, Alan Gutierrez, Jillian L. Astarita, Minh Thai, Jeffrey Hung, Yeqing Angela Yang, Tony Pourmohamad, Patricia Himmels, Marco De Simone, Justin Elstrott, Aude-Hélène Capietto, Rafael Cubas, Zora Modrusan, Wendy Sandoval, James Ziai, Stephen E. Gould, Wenxian Fu, Yulei Wang, James T. Koerber, Shomyseh Sanjabi, Ira Mellman, Shannon J. Turley, Sören Müller

**Affiliations:** Genentech, South San Francisco, CA USA

**Keywords:** Cancer microenvironment, Tumour immunology, Tumour immunology

## Abstract

TGFβ signaling is associated with non-response to immune checkpoint blockade in patients with advanced cancers, particularly in the immune-excluded phenotype. While previous work demonstrates that converting tumors from excluded to inflamed phenotypes requires attenuation of PD-L1 and TGFβ signaling, the underlying cellular mechanisms remain unclear. Here, we show that TGFβ and PD-L1 restrain intratumoral stem cell-like CD8 T cell (T_SCL_) expansion and replacement of progenitor-exhausted and dysfunctional CD8 T cells with non-exhausted T effector cells in the EMT6 tumor model in female mice. Upon combined TGFβ/PD-L1 blockade IFNγ^hi^ CD8 T effector cells show enhanced motility and accumulate in the tumor. Ensuing IFNγ signaling transforms myeloid, stromal, and tumor niches to yield an immune-supportive ecosystem. Blocking IFNγ abolishes the anti-PD-L1/anti-TGFβ therapy efficacy. Our data suggest that TGFβ works with PD-L1 to prevent T_SCL_ expansion and replacement of exhausted CD8 T cells, thereby maintaining the T cell compartment in a dysfunctional state.

## Abstract (web summary)

It has been previously shown that combining immune checkpoint inhibitors with TGFβ blockade potentiates anti-tumor immune responses. Here the authors show that, in an immune excluded preclinical tumor model, combining therapeutic anti-PD-L1 with anti-TGFβ treatment promotes expansion and differentiation of stem-cell like CD8 + T cells.

## Introduction

Cancer immunotherapy holds great promise for improving patients’ survival and quality of life. Nevertheless, even in cancer types sensitive to PD-1/PD-L1 blockade, only 25–35% of patients achieve complete and durable responses^1^. To understand the parameters governing immunotherapy response and resistance, a framework of three tumor-immune phenotypes has been established based on histopathology: (I) inflamed, in which CD8 T cells infiltrate the tumor parenchyma (II) excluded, in which CD8 T cells accumulate at the tumor–stroma boundary and (III) desert, in which CD8 T cells are mainly absent^2,3^. Patients with inflamed tumors have the best overall prognosis compared to those with desert or excluded tumors, which represent ~50% of all human tumors^2,4^.

In melanoma, head and neck, and gastric cancer, patients with inflamed tumors respond favorably to anti-PD-1 therapy and show higher expression of T cell activation markers, *IFN*γ and IFNγ signaling genes compared to non-responders^5^. Similar results were found in urothelial bladder and non-small cell lung cancer patients who benefit from anti-PD-L1 antibodies^6–8^. Despite the presence of CD8 T cells, excluded tumors show increased resistance to checkpoint blockade compared to patients with inflamed tumors^8–11^. Excluded tumors are typically characterized by elevated transforming growth factor beta (TGFβ) signaling. Combining checkpoint blockade with inhibitors of the immunosuppressive cytokine TGFβ potentiates curative, CD8 T cell-dependent anti-tumor immunity both in immune-excluded^8,12,13^ and other preclinical models^12,14–20^. These promising preclinical findings have inspired multiple clinical trials^21^ of therapeutic agents that target TGFβ^16,19,22^ in combination with checkpoint inhibition. Unfortunately, effective blockade of all three TGFβ isoforms may lead to unintended systemic side effects, such as tumor induction, cardiovascular and bleeding toxicities^23^, tissue inflammation, and autoimmunity. Moreover, the currently limited clinical success of TGFβ inhibitors reflects a need to further understand the impact of combined inhibition of TGFβ and checkpoint blockade agents such as PD-1/PD-L1 on the various cell types in the TME.

Even though CD8 T cell infiltration allows retrospective prediction of responsiveness to checkpoint blockade, it remains incompletely understood how specific therapies, including TGFβ blockade, may impact the origin and fate of CD8 T cells. Chronic antigen exposure in cancer and other diseases induces T cells to enter an exhausted/dysfunctional state characterized by gradual loss of effector functions and increased levels of co-inhibitory receptors such as LAG3, TIM3, PD-1, and TIGIT^24,25^. This imprinting in T-exhausted cells cannot be overcome by checkpoint blockade^26^. Several studies conducted in chronic viral infection mouse models showed that T-exhausted cells originate from TCF1^+^ PD-1^+^ stem-like cells^27–30^. Recent work in cancer identified two subsets of TCF1/7^+^ stem-like CD8^+^ memory T cells, putative T-memory progenitors, with distinct fate commitments. TCF1/7^+^ progenitors with low levels of PD-1 expression were committed to a functional phenotype (T stem cell-like, T_SCL_), whereas TCF1/7^+^ progenitors expressing high levels of PD-1 were committed to a dysfunctional, exhausted state and thus named T progenitor-exhausted or T_PEX_^31,32^. T_SCL_ have been shown to occupy specific niches in the TME^33^ and are required for tumor control in response to immunotherapy^34–36^. Several human studies have similarly highlighted the importance of TCF1^+^ antigen-experienced T cells within the TME as critical responders to checkpoint blockade^36–38^. Effector memory cells, co-expressing *Tcf7* and *Cxcr3* as well as granzymes (most prominently *Gzmk*), with low to intermediate expression of *Pdcd1* and low levels of clonal expansion resemble the T_SCL_ population in mice^39^. They may similarly give rise to diverse cytotoxic T-cell expression phenotypes.

In chronic viral infections, TGFβ signaling is essential for T_PEX_ maintenance^40^ and elevated in exhausted T cells^41^. Knocking out endogenous TGFβ receptor II (TGFBR2) in CAR T cells prevented the exhaustion of CARs^42^. CD8 T cell-intrinsic TGFβ signaling has further been described to suppress CD8 T cell-dependent tumor rejection by limiting their trafficking from the tumor-draining lymph node (dLN) into the tumor^43^. If and how blocking TGFβ affects CD8 T cell heterogeneity in the TME remains largely unclear. In particular, the impact of combining anti-PD-L1 with anti-TGFβ treatment on progenitor and effector CD8 T cells and their interactions with other key populations in the TME is not well understood.

In this study, we use a multi-omics approach to unravel how PD-L1 and TGFβ affect CD8 T cell phenotypes and impact tumor cells, myeloid cells, and fibroblasts. Our data demonstrate that dual blockade induces a shift in the balance of dysfunctional and progenitor cells with a reduction in T_PEX_ and an expansion of clonally diverse stem-like (T_SCL_) CD8 T cells. Bioinformatic inference based on scRNA-seq analysis of tumor-infiltrating CD8 T cells suggests that T_SCL_ can give rise to IFN-producing effector progeny, and their increase is accompanied by TME-wide interferon licensing. Moreover, we show that TGFβ prevents T-effector cell (T_EFF_) replenishment by T_SCL_ in the CD8 T cell compartment and demonstrate that interferon licensing throughout the TME is associated with better overall survival in patients.

## Results

**Fig. 1:**
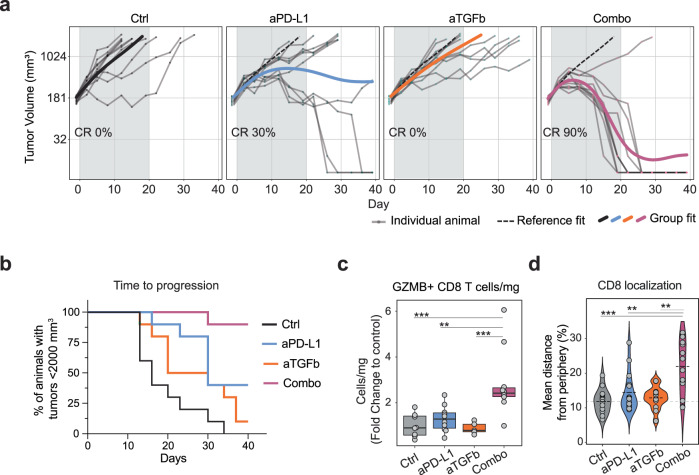
TGFβ restrains the anti-tumor response induced by anti-PD-L1 in the TME. **a** Tumor volume (*y* axis) of EMT6 tumors treated with anti-PD-L1 and anti-TGFβ alone or in combination over time (*x* axis). Grey shade indicates treatment duration. Individual animal curves (grey lines) and group fit curves (thick solid lines) of the control group (black) and treatment groups (colored) are provided. CR complete responder. Representative experiment (*n* = 10 for all groups). **b** Percentage of animals bearing EMT6 tumor smaller than 2000 mm^3^ in the same study as (**a**) (*y* axis) over time (*x* axis). Representative experiment (*n* = 10 for all groups). **c** Tumor GZMB^+^ CD8 T cell quantification via flow cytometry (cells per mg of tissue, fold change over the average of the control group on the *y* axis; groups on the *x* axis; data from two independent experiments, *n* = 10 for all groups). **d** Quantification of tumor-infiltrating CD8 T cell localization by immunohistochemistry (*y* axis: % of the distance from tumor periphery; *x* axis: groups; two independent experiments, *n* = 15 for all groups; the dotted line represents mean). Source data are provided as a Source Data file. Ctrl = control (anti-GP120), aPD-L1 = anti-PD-L1, aTGFb = anti-TGFβ, combo = combination anti-PD-L1^+^ anti-TGFβ; adj *P* values *<0.1, **<0.05, ***<0.01, ****<0.001 Dunn’s test (two-sided) with Benjamini–Hochberg multiple testing correction. Adjusted *P* values for (**c**): 0.00136 (ctrl vs combo), 0.0166 (aPD-L1 vs combo), 0.00334 (aTGFb vs combo). Adjusted *P* values for (**d**): 0.00262 (ctrl vs combo), 0.0189 (aPD-L1 vs combo), 0.0258 (aTGFb vs combo). **c** Whiskers represent the minimum and maximum (unless points extend 1.5 * IQR from the hinge, then shown as individual points), the box represents the interquartile range, and the center line represents the median.

Consistent with previous work^8,13,44^, we found that combining therapeutic anti-PD-L1 with anti-TGFβ treatment (a monoclonal antibody that neutralizes all three active TGFβ isoforms) promoted tumor regression and survival in the immune-excluded EMT6 tumor model compared to control and single agent treatments (70% or more complete responders in combination treatment compared to ~30% in anti-PD-L1, Fig. 1a, b and Supplementary Fig. 1A). In addition, at 7 days post initiation of therapy and before any change in tumor volume, activated GZMB^+ ^CD8^+^ T cell number (Fig. 1c) and overall CD8 T cell infiltration into the tumor increased in combination therapy (Fig. 1d and Supplementary Fig. 1B, C).

### Single-cell RNA-seq identifies T_SCL_ and T_PEX_ CD8 T cells in EMT6 tumors

**Fig. 2:**
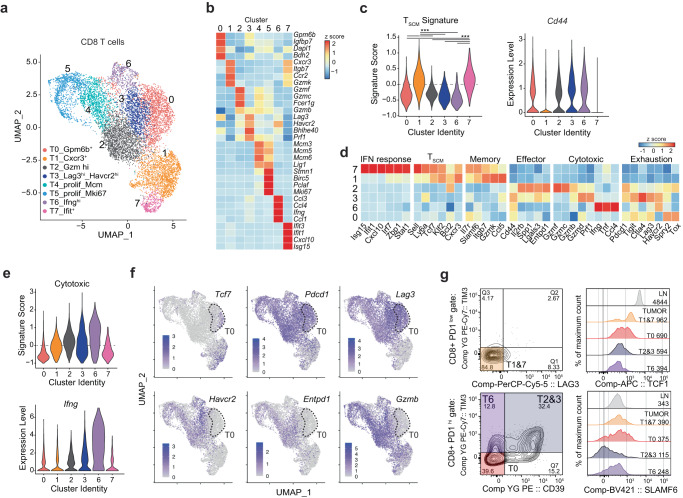
Single-cell RNA-seq identifies T_SCL_ and T_PEX_ CD8 T cells in EMT6 tumors. **a** UMAP of 9,525 CD8 T cells (dots) colored by cluster (*n* = 5 mice per group). **b** Heatmap of relative average expression of four marker genes in each cluster from (**a**). **c** Scores for T_SCM_ signature and *Cd44* expression in cells from clusters in (**a**). ***Pairwise Wilcoxon rank-sum test (two-sided) *P* < −7.18e- 95. **d** Heatmap of relative average expression of selected genes in indicated clusters from (**a**). **e** Cytotoxic signature enrichment scores (top; all pairwise two-sided Wilcoxon rank-sum tests *P* < 0.004) and *Ifng* expression (bottom) in clusters from (**a**). **f** Levels of expression [Log(CPM/100 + 1)] of selected genes in UMAP space. The dotted line highlights cluster 0 cells. **g** Flow cytometry analysis of CD8 T cells. Left: gating strategy (representative plots of untreated sample, *n* = 3). Right: TCF1 and SLAMF6 expression in cells populating cluster T0 (PD1^hi^LAG3^+^TIM3^−^), T1&7 (PD1^low^LAG3^−^), T2&3 (PD1^hi^LAG3^+^TIM3^+^CD39^+^), and T6 (PD1^hi^LAG3^+^TIM3^+^CD39^-^) compared to CD8^+^ T cells from naive lymph node (LN). Numbers indicate mean fluorescence intensity.

We next performed scRNA-seq on day seven after treatment initiation and before changes in tumor volume to define the early impact of combination treatment on intratumoral CD8 T cells at high resolution (Supplementary Fig. 1D). We identified eleven clusters among 25,063 sorted TCRb^+^ T cells (Supplementary Table 1). Based on the expression of established markers, they broadly classified into naive T cells (*Lef1*^+^), NKT cells (*Ncr1*^+^), CD8^+^ cells (*Cd8a*^+^), T helper cells (*Cd4*^+^), T regulatory cells (*Foxp3*^+^) and proliferating cells (*Mki67*^+^) (Supplementary Fig. 2A, B and Supplementary Data 1). An in-depth analysis of the non-naive CD8 T cell compartment revealed eight transcriptional clusters (Fig. 2a, b). To characterize each of these CD8 T cell clusters, we compared their gene expression profiles to known transcriptional markers of T cell function.

Clusters T1 and T7 showed high expression of memory T cells genes (*Ccl5, Zfp36l2, Cxcr3, Gpr183*^45–52^) and exhibited elevated expression of a T stem cell memory (T_SCM_) signature (*Tcf7, Sell, Il2rb, Cxcr3, Ly6a, Bcl2* and *Klf2*^53–57^), but lacked expression of *Cd44* (Fig. 2c and Supplementary Fig. 2C). T1/T7 cells were distinct from naive CD8^+^ T cells as they expressed activation genes (*Cd69, Ctla4, Tigit, Pdcd1*, Supplementary Fig. 2D). While T1 and T7 shared expression of many genes including *Tcf7*, T7 exhibited a distinct IFN response signature (*Isg15, Ifits, Stat1, Zbp1*, and *Irf7*) (Fig. 2d and Supplementary Fig. 2D), similar to human and mouse T_SCM_^58–60^. Moreover, compared to other clusters, T1/T7 expressed lower levels of effector, cytotoxic, and exhaustion genes, such as *Pdcd1*, *Entpd1*, *Gzmb*, and *Prf1*. Given their transcriptional profile, we classified T1 and T7 as T stem cell-like cells (T_SCL_).

T2 and T3 showed higher expression of effector/cytotoxicity markers, as well as genes encoding immune checkpoint molecules (such as *Pdcd1, Havcr2, Ctla4*), whereas T6 and T0 were characterized by the expression of co-inhibitory receptors and immune checkpoints (*Lag3*, *Tigit*) (Fig. 2d). While cluster T0 expressed the highest levels of *Tox*, cluster T6 expressed the highest levels of *Ifng, Tnf*, and *Ccl4*. Signatures of T effector^8^, exhausted^61^ and cytotoxic T cells (Supplementary Table 2) were mainly enriched in T2, T3, and T6, suggesting the presence of effector cells at different stages of differentiation, including exhaustion (Fig. 2e and Supplementary Fig. 2E). T6 expressed markedly higher levels of *Ifng* than any other CD8 T cell cluster, and anti-PD-L1 and combination treatments elevated its expression (Fig. 2e and Supplementary Fig. 2F).

T0 had the lowest score for the cytotoxic signature and expressed genes associated with TGFβ signaling (*Timp2*, *Gpm6b*, lack of *Gzmb*)^62–67^, deletional tolerance^68,69^ and the contraction phase of the T cell activation cycle^70–75^ (Fig. 2f, Supplementary Fig. 2E, G, and Supplementary Data 1). Similar to T precursor exhausted (T_PEX_) cells previously described in tumors^76^, T0 showed high expression of *Pdcd1* and *Lag3*, and a subset expressed *Tcf7* and *Slamf6* but lacked expression of *Havcr2* and *Entpd1*. These observations point to *Gpm6b*^+^ cells in T0 as T_PEX_ (Fig. 2f and Supplementary Fig. 2H, I). Cells in T0 were further characterized by the expression of *Nt5e* (Supplementary Fig. 2G), previously described as an indicator of TGFβ signaling in T cells^66,77^ and as a marker of T_PEX_ in chronic infection^40^.

Flow cytometry (Supplementary Fig. 3A) validated the highest expression of TCF1 in T1/T7 cells (CD8^+^PD-1^low^LAG3^−^TIM3^−^), followed by T0 (CD8^+^PD-1^hi^LAG3^+^TIM3^−^CD39^−^), while effector cells T2/T3 (CD8^+^PD-1^hi^LAG3^+^TIM3^+^CD39^+^) and T6 (CD8^+^PD-1^hi^LAG3^+^TIM3^+^CD39^−^) were negative for TCF1 staining (Fig. 2g). SLAMF6 expression was similarly consistent with our measurements in scRNA-seq data, thus validating our single-cell clusters on the protein level (Fig. 2g). Based on this analysis, we defined PD-1, LAG3, and TIM3 as our core markers to gate T_SCL_ (PD-1^low^LAG3^−^TIM3^−^) and T_PEX_ (PD-1^hi^LAG3^+^TIM3^−^) by flow cytometry (Supplementary Fig. 3B).

### Dual blockade of TGFβ and PD-L1 increases the abundance of T_SCL_ at the expense of T_PEX_ CD8 T cells

**Fig. 3:**
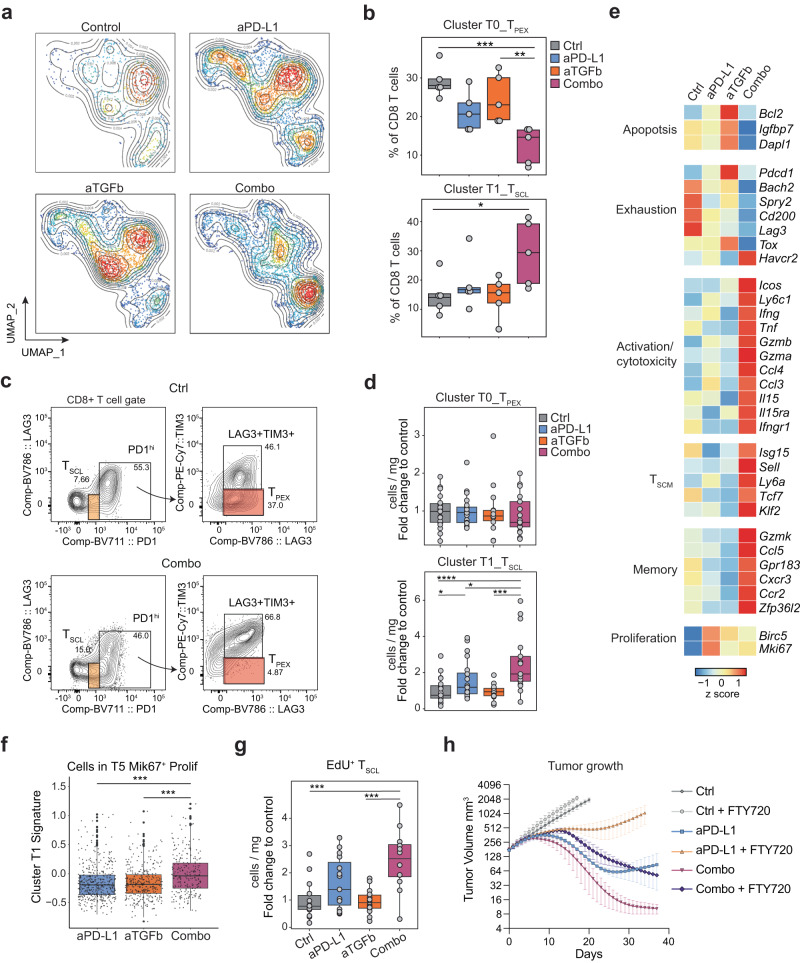
Combined PD-L1 and TGFβ blockade allows T_SCL_ expansion in the tumor. **a** UMAP as in Fig. 2a, here colored by cell density. Red indicates high cell density, blue low density. **b** Quantification of the frequency of cells in cluster T0 and T1 (*y* axis; *n* = 5 per group of treatment from one experiment) in each animal (dots) by treatment group (*x* axis). **c** Gating strategy for flow cytometry analysis of CD8 T cells at day 7 after initiation of treatment. Representative plots of control and combo samples. **d** Flow cytometry quantification of CD8 T_SCL_ (PD1^low^LAG3^-^) and T_PEX_ cells (PD1^hi^LAG3^+^TIM3^-^) (cells per mg of tissue, fold change over average of the control group: *y* axis; groups: *x* axis; data from three independent experiments, ctrl *n* = 19, aPD-L1 *n* = 20, aTGFb *n* = 14, combo *n* = 17). **e** Heatmap of relative average expression of selected genes in CD8 T cells for each treatment group. **f** Signature score in cells (dots) from cluster T5 based on genes enriched in cluster T1. *P* value is from paired Wilcoxon rank-sum tests (two-sided) comparing between treatment conditions. Control not included due to low number of cells in cluster T5. (aPD-L1 *n* = 420 cells, aTGFb *n* = 326 cells, Combo *n* = 284 cells; all conditions include five animals). **g** Flow cytometry quantification of EdU^+^ T_SCL_ (cells per mg of tissue, fold change over average of the control group: *y* axis; groups: *x* axis; data from two independent experiments, ctrl *n* = 14, aPD-L1 *n* = 15, aTGFb *n* = 14, combo *n* = 12). **h** Group fit curves of EMT6 tumor growth treated with FTY720 in addition to control, anti-PD-L1 and combo. Two independent experiments (10 animals in each experiment, mean ± SEM (see Supplementary Fig. 4i for individual animal curves)). Source data are provided as a Source Data file. Ctrl = control (anti-GP120), aPD-L1 = anti-PD-L1, aTGFb = anti-TGFβ, combo = combination anti-PD-L1 + anti-TGFβ; Adj *P* values *<0.1, **<0.05, ***<0.01, ****<0.001 Dunn’s test (two-sided) with Benjamini–Hochberg multiple testing correction. Adjusted *P* values for (**b**): 0.005517513 (ctrl vs combo T0), 0.030889647 (aTgfb vs combo T0), 0.08364055 (ctrl vs combo T1). Adjusted *P* values for (**d**): 0.000512 (ctrl vs combo), 0.0679 (aPD-L1 vs combo), 0.00197 (aTGFb vs combo), 0.0841 (ctrl vs aPD-L1). Adjusted *P* values for (**g**): 0.00347 (ctrl vs combo), 0.00308 (aTGFb vs combo). **b**, **d**, **f**, **g** Whiskers represent the minimum and maximum (unless points extend 1.5 * IQR from the hinge, then shown as individual points), the box represents the interquartile range, and the center line represents the median.

To understand if CD8 T cell subsets are affected by TGFβ and/or PD-L1 blockade, we compared their abundance between treatment conditions. While cluster T5 increased and T6 decreased in single (but not in combination) treatments, dual blockade significantly changed the abundance of cells from two of the eight clusters. We found that cluster T1 (*Cxcr3*^+^ T_SCL_) expanded 2.0-fold and cluster T0 (*Gpm6b*^+^ T_PEX_) contracted 2.3-fold, whereas neither single agent treatment significantly affected these populations (Fig. 3a, b, Supplementary Fig. 4A, and Supplementary Table 3). We confirmed the relative T_SCL_ increase and T_PEX_ decrease in established tumors following dual TGFβ and PD-L1 blockade by flow cytometry (Fig. 3c and Supplementary Fig. 4B). The number of T_PEX_ and T_SCL_ per mg of tumor tissue suggested that the overall effect of the anti-PD-L1/ TGFβ combination is an increase in the total number of T_SCL_ in the TME (Fig. 3d).

Computational inference of cluster abundance in independent cohorts of mice substantiated the changes in cluster T1 frequency both at the transcriptional (bulk RNA-seq deconvolution of sorted T cells) and translational (Hyper Reaction Monitoring Mass Spectrometry of whole tumor tissue) levels (Supplementary Fig. 4c–e and Supplementary Data 2). Differential expression analysis on CD8 T cells revealed that anti-TGFβ monotherapy can downregulate the expression of a subset of exhaustion markers such as *Lag3* and *Cd200*. Combination therapy reduced their expression further and repressed a broader set of exhaustion genes, including *Tox* (highly expressed by T_PEX_). The combination also increased the expression of stem-like and cytotoxic genes such as *Ly6a*, *Gzmb*, and *Ifng* (Fig. 3e and Supplementary Data 3). While anti-TGFβ monotherapy increased the expression of genes associated with apoptosis (such as *Bcl2*, and *Igfbp7*), the combination therapy had the opposite effect.

To investigate if the expansion of T_SCL_ cells could be due to intratumoral in situ proliferation, we scored the cells in the proliferating cluster T5 for a gene set enriched in T_SCL_ cluster T1. The significant enrichment for this score in the combination suggests that T_SCL_ can expand in situ and that this process is more prevalent with combination therapy (Fig. 3f). A 24-h 5-ethynyl-2’deoxyuridine (EdU) pulse experiment further substantiated the increased proliferation of T_SCL_ cells with combination treatment, as shown by the significant increase in EdU^+^ T_SCL_ cells found in the tumor (Fig. 3g and Supplementary Fig. 4F–G). To more directly test the possibility that T_SCL_ cells already present in the tumor at the time of TGFβ and PD-L1 blockade have the potential to control tumor growth, mice were treated with FTY720 (daily for three weeks starting when tumors reached ~150 mm^3^) to block egress of T cells from lymph nodes and prevent tumor infiltration by new circulating cells. Flow cytometric analysis confirmed that the blood of FTY720-treated mice was devoid of circulating T cells at days 7 and 14 (Supplementary Fig. 4H). While FTY720 greatly reduced the response seen with anti-PD-L1 alone, ~50% of mice still exhibited complete tumor regression in combination with anti-TGFβ (Fig. 3h and Supplementary Fig. 4I). These data suggest that CD8 T cells already present in the TME at the time of treatment onset have, at least in a subset of animals, the potential to drive curative anti-tumor immunity.

### Velocity and TCR analyses identify T_SCL_ as precursors of diverse transcriptional states, including IFNγ^hi^ effector T cells

**Fig. 4:**
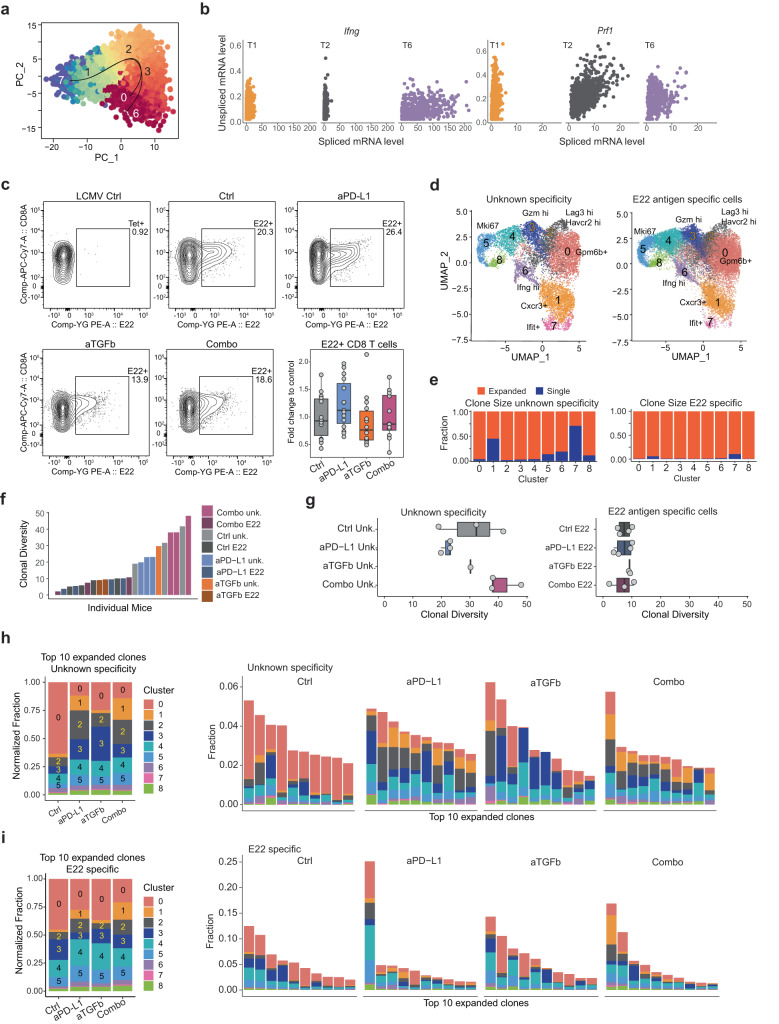
T_SCL_ are precursors of diverse transcriptional states. **a** Cells as in Fig. 2a, here in PCA space and colored by pseudo time. Trajectory is given by smoothed curves. **b** Quantification of spliced vs unspliced *Ifng* (left) and *Prf1* (right) expression in cells colored by cluster from (**a**) for clusters T1, T2, and T6. **c** Flow cytometry analysis of E22-specific CD8 T cells at day 7 after initiation of treatment. Representative plots with flow cytometry control performed using an irrelevant tetramer (LCMV Ctrl). At the bottom right: quantification of the E22-specific CD8 T cells in the four groups of treatments (% of E22^+^ cells in the CD8 T cells, fold change over average of the control group: *y* axis; groups: *x* axis; data from two independent experiments, Ctrl *n* = 14; aPD-L1 *n* = 15; aTGFb *n* = 14; Combo *n* = 12). **d** (Left) UMAP of 10,521 CD8 T cells (dots) of unknown specificity from a single-cell RNA/TCR experiment colored by cluster. (Right) UMAP of 25,005 CD8 T cells specific for E22 in the same UMAP space as on the left and colored by cluster membership. **e** Quantification of the fraction of cells in each cluster that are either single TCR clones or expanded (>1 cell with clonotype) for unknown specificity (left) and E22-specific cells (right). **f** Clonal diversity as mean Hill diversity index (*y* axis) per mouse (bar) for unknown specificity and E22-specific cells. Mice are ranked by clonal diversity (*x* axis). **g** Distribution of clonal diversity (*x* axis) per animal for unknown specificity (left; Ctrl *n* = 3, aPD-L1 *n* = 3, aTGFb *n* = 1, Combo *n* = 3) and E22-specific cells (right; Ctrl *n* = 4; aPD-L1 *n* = 4; aTGFb *n* = 2; Combo *n* = 3) for each of the four treatment groups. **h** (Left) Fraction of cells assigned to a particular transcriptional cluster based on CD8 T cells from the 10 most expanded clones in each treatment group. (Right) Fraction of CD8 T cells assigned to each of the 10 most expanded clones in every group (one bar per clone) colored by transcriptional cluster membership. Both figures for T cells with unknown specificity. **i** Same as in (**h**) but for E22-specific cells. Source data are provided as a Source Data file. Ctrl = control (anti-GP120), aPD-L1 = anti-PD-L1, aTGFb = anti-TGFβ, combo = combination anti-PD-L1 + anti-TGFβ. **c**, **g** Whiskers represent the minimum and maximum (unless points extend 1.5 * IQR from the hinge, then shown as individual points), the box represents the interquartile range, and the center line represents the median.

Next, we were interested in the developmental relationships between T cell subsets. Trajectory analysis underlined the possibility of a stem/memory nature of *Cxcr3*^+^ T_SCL_ clusters T7 and T1, positioning T_SCL_ cells at the beginning of a transcriptional trajectory, *Gpm6b*^+^ T_PEX_ (T0) and *Ifng*^hi^ T_EFF_ (T6) cells as potential endpoints, and T_EFF_ (T2/T3) in between (Fig. 4a and Supplementary Fig. 5A). The progenitor-stem-like phenotype of cells in T1/T7 was additionally supported by RNA velocity analysis, an additional method to predict the future state of individual cells from scRNA-seq data^78^. While cells in T1/T7 expressed low levels of mature spliced RNAs encoding *Ifng* and *Prf1*, the expression levels of both *Ifng* and *Prf1* pre-mRNA were high. This suggests cells in cluster T1/T7 were poised to transition towards more activated clusters that show higher amounts of spliced RNA for these two genes (Fig. 4b and Supplementary Fig. 5B).

The classification of T1 and T7 as T_SCL_ and the transcriptional similarity between T_SCL_ and naive T cells raised the possibility that these two clusters might have a diverse T cell receptor (TCR) repertoire, as previously reported for T_SCM_^79^. To test this hypothesis, we performed combined single-cell RNA and TCR-seq on CD8 T cells—with either unknown antigen specificity and E22 antigen specificity (an EMT6 specific mutation that generates an MHCI-restricted immunogenic neo-antigen^80^)—sorted from tumors following the same four therapeutic antibody treatments. E22 antigen-specific T cells represented an average of ~20% of the CD8^+^ PD-1^+^ cells in the tumor, and none of the treatments significantly changed their frequency (Fig. 4c). The identified scRNA/TCR-seq clusters showed strong transcriptional overlap with the initially presented scRNA-seq data (Fig. 4d, Supplementary Fig. 5C, and Supplementary Data 4). We further observed similar frequency changes in T0 and T1 (for cells with unknown and E22 specificity) and similar RNA velocity observations (Supplementary Fig. 5D–G). Notably, in the unspecific dataset, the TCR analysis demonstrated that T1 and T7 represented the most clonally diverse population of CD8 T cells (Fig. 4e), thus further supporting their stem-like classification. This trend was also observed in the E22-specific dataset, although to a much lesser degree.

In contrast, cells in T0 showed a stronger degree of clonal expansion, consistent with their annotation as T_PEX_ (Fig. 4e and Supplementary Fig. 5H). By restricting the pool of clones to a single antigen, we identified a decreased clonal diversity in E22 enriched cells compared to not enriched cells (Fig. 4f). When comparing clonal diversity between conditions in antigen-specific cells or cells with unknown specificity, we did not observe any significant differences between the treatment groups (Fig. 4g).

Distribution analysis of the ten most expanded clones highlighted further differences between treatments: in controls, the majority of expanded clones were found in T_PEX_ cluster T0 (Fig. 4h, i), whereas in combination treatment, expanded clones were present across all clusters, with T1 representing ~20% of all CD8 T cells (Fig. 4h, i) in both E22-specific and non-specific datasets. This suggests that the expanded T cells exhibit an increased diversity of transcriptional states in combination treatment.

In summary, T_SCL_ cells expand in tumors following combination therapy at day 7 post-treatment initiation. They may give rise to IFNγ^hi^ T_EFF_, while the relative abundance of T_PEX_ decreases. In addition, T_SCL_ shows an increased clonal diversity and proliferate in the tumor, underlining their potential to fuel the anti-tumor response in combination treatment.

### Cross-compartmental IFNγ licensing accompanies combination treatment-induced TSCL increase

To understand if cell-extrinsic changes might additionally mediate the expansion of T_SCL_ in the combination, we probed how tumor cells, fibroblast, and myeloid cells can communicate with T cells in the TME in our scRNA-seq data.

**Fig. 5:**
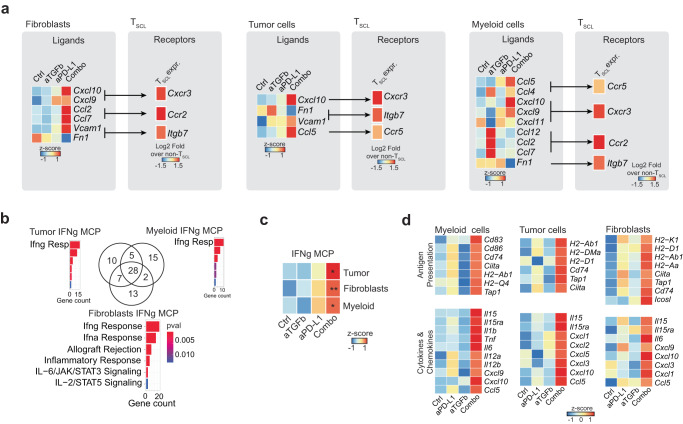
Combination treatment induces an IFNγ response program in the TME. **a** Relative average expression in each treatment condition for ligands (left) predicted to bind T_SCL_-specific receptors (right; Log2FC of T_SCL_ vs all other T cell subsets); for fibroblast, tumor cell as well as myeloid interactions separately. **b** Venn diagram comparing the 50 top genes of the IFNg MCP program in fibroblasts, myeloid cells, and tumor cells. Numbers indicate the number of shared genes between cell types. Pathway enrichment analysis results are given by bar plots, where color indicates the *P* value (two-sided Fisher exact test) and bar height represents the number of genes overlapping with the respective pathway. **c** IFNg MCP program activity by treatment conditions and cell type. *P* values are from the comparison of each treatment to the control group (*n* = 5 per group). Adjusted *P* values: 0.009673458 (fibroblasts), 0.03598864 (myeloid), 0.03598864 (tumor). **d** Heatmap of relative average expression of indicated genes across conditions. Ctrl = control (anti-GP120), aPD-L1 = anti-PD-L1, combo = combination anti-PD-L1 + anti-TGFβ. Adj *P* values *<0.05, **<0.01, Dunn’s test (two-sided) with Benjamini–Hochberg multiple testing correction.

Our analysis of cells sorted from EMT6 tumors (Supplementary Fig. 1D) revealed that the combination therapy reduced the expression of TGFβ-inducible CAF markers in fibroblasts (Supplementary Fig. 6A, top). Furthermore, we found both iCAFs and myCAFs transition into an interferon response state in the combination treatment. We did not find a loss of a particular subset of malignant cells but downregulation of individual genes across tumor cell clusters in a pseudobulk differential expression analysis (Supplementary Fig. 6A, middle). In the myeloid compartment, the combination treatment enriched for monocytes rather than macrophages (Supplementary Fig. 6A, bottom). We next predicted interactions between T_SCL_ and tumor, stromal and myeloid compartments (Fig. 5a). Our analysis revealed that combination treatment induces the expression of ligands in non-T cells that bind receptors specifically expressed by T_SCL_. These ligands, such as CXCR3 ligands CXCL9/10, can act on TSCL by either increasing their migration or retaining them in the TME^81^. CXCL9 was identified as an essential mediator of the efficacy of the anti-αvβ8 integrin anti-PD-1 combination therapy in the EMT6 model^82^. Given many of these ligands have been described as interferon-inducible, we hypothesized that their expression might be part of an interferon response across the multiple cell compartments.

To test this hypothesis, we detected expression programs underlying common cellular activities (Supplementary Data 5) in single-cell RNA-seq data across all four conditions and all major non-T cell compartments of the TME: tumor cells, myeloid cells, and fibroblasts. We identified a program enriched in the “Interferon-gamma response” MsigDB Hallmark gene set in all three compartments (Fig. 5b and Supplementary Fig. 6B). We termed this IFNγ MCP. Quantification of the IFNγ MCP program in all animals revealed a significant enrichment of IFNγ MCP program activity following combination treatment in tumor cells, myeloid cells, and CAFs (Fig. 5c). Anti-PD-L1 monotherapy also showed a trend of upregulation of this program. Nevertheless, the combination with TGFβ blockade amplified the signal, and we observed a significant induction compared to the control in each of the three compartments. Furthermore, proteomic analysis validated the IFNγ MCP program activation (Supplementary Fig. 6C). Similarly, pathway analysis of significantly upregulated genes in pooled pseudobulk samples comparing combination-treated mice to control (Supplementary Fig. 6A (right) and Supplementary Data 6 and  7) revealed a striking positive enrichment of multiple interferon-inducible inflammatory signaling (*Cxcl9, Cxcl10, Il15, Il15ra, Tnf*) and antigen presentation pathways (*H2-Ab1, Tap1, Cd74*) in the tumor, myeloid and fibroblast compartments (Fig. 5d).

### IFNγ and intact IFNγ receptor signaling in tumor cells are required for a complete response to the combination

**Fig. 6:**
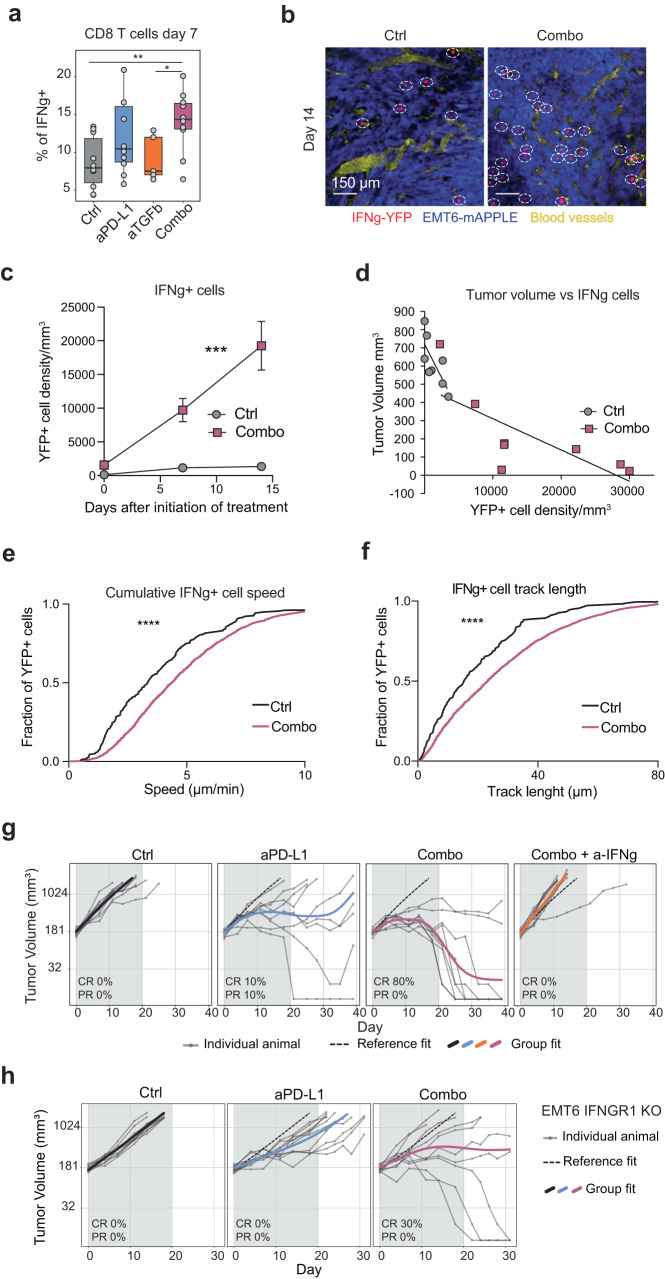
Combination treatment increases the amount of IFNγ^+^ CD8 T cells and their mobility in the TME. **a** Flow cytometry quantification of IFNγ^+^ CD8 T cells (ctrl *n* = 10, aPD-L1 *n* = 10, aTGFb *n* = 5, combo *n* = 10). **b** Representative images of EMT6-mAPPLE tumors at 14 days after initiation of treatment (*n* = 8 per group). Blue, tumor cells; Red, IFNγ-YFP^+^ cells; Yellow, blood vessels; white dashed circles highlight IFNγ-YFP cells. **c** Density of IFNγ-YFP^+^ cells at three time points after initiation of treatment (*n* = 8 Ctrl, *n* = 7 combo (non-responders excluded); *P* < 0.0001 two-way ANOVA, mixed-effect analysis; mean and SEM are shown). **d** Correlation between tumor volume (*y* axis) and IFNγ-YFP^+^ cells density (*x* axis) (*n* = 8 per group). **e** IFNγ-YFP^+^ cell speed at day 7–9 after initiation of treatment (Control: *n* = 179 cells from 8 mice; Combination: *n* = 2310 cells from 7 mice (non-responders excluded); *P* < 0.0001, two-tailed Kolmogorov–Smirnov test). **f** IFNγ-YFP^+^ cells track length at day 7–9 after initiation of treatment (Control: *n* = 179 cells from 8 mice; Combination: *n* = 2,310 cells from seven mice (non-responders excluded); *P* value < 0.0001, two-tailed Kolmogorov–Smirnov test). **g** Tumor volume (*y* axis) of EMT6 tumors treated with anti-PD-L1, combo or combo plus IFNγ neutralizing antibody (a-IFNg) over time (*x* axis). Grey shade indicates treatment duration. Individual animal curves (grey lines) and group fit curves (thick solid lines) of the control group (black) and treatment groups (colored) are provided. CR complete responder, PR partial responder. Representative experiment of three (*n* = 10 for all groups). **h** Tumor volume (y axis) of EMT6 IFNGR1 KO tumors treated with anti-PD-L1 or combo over time (*x* axis). Grey shade indicates treatment duration. Individual animal curves (grey lines) and group fit curves (thick solid lines) of the control group (black) and treatment groups (colored) are provided. CR complete responder, PR partial responder (*n* = 10 for all groups). Source data are provided as a Source Data file. Ctrl = control (anti-GP120), aPD-L1 = anti-PD-L1, aTGFb = anti-TGFβ, combo = combination anti-PD-L1^+^ anti-TGFβ; Adj *P* values *<0.1, **<0.05 Dunn’s test (two-sided) with Benjamini–Hochberg multiple testing correction unless differently specified. Adjusted *P* values for (**a**): 0.0382 (ctrl vs combo), 0.0851 (aTGFb vs combo). **a** Whiskers represent the minimum and maximum (unless points extend 1.5 * IQR from the hinge, then shown as individual points), the box represents the interquartile range, and the center line represents the median.

In line with our findings that T_SCL_ are present in the tumor, expand in response to combination therapy before tumor regression, and can give rise to several T_EFF_ cell subsets, we observed an increase in the percentage of IFNγ^+^ CD8 T cells in combination-treated tumors compared to control and anti-TGFβ treatment by flow cytometry (Fig. 6a). Across all populations sorted from EMT6 tumors, CD8 T cells showed the highest level of expression of IFNγ, pointing to CD8 T cells as the primary source of IFNγ in the TME (Supplementary Fig. 7A–C). Therefore, we hypothesized that IFNγ^hi^ T_EFF_ cells mediate the observed TME interferon licensing and thus characterize their localization, quantity, and movement. We used intravital two-photon microscopy in an IFNγ-YFP reporter mouse inoculated with EMT6 tumor cells (Supplementary Fig. 7D, E). This allowed us to image the locations, velocities, and path lengths of individual IFNγ^+^ T cells within tumors. We found that combination treatment induced increased IFNγ^+^ T cell accumulation within the tumor center compared to control treatment (Fig. 6b, c and Supplementary Fig. 7F). The number of IFNγ^+^ T cells was inversely correlated with tumor volume (Fig. 6d), consistent with their presence promoting tumor clearance. IFNγ^+^ T cells in combination-treated tumors showed increased speeds and greater total path lengths compared to IFNγ^+^ T cells in control-treated tumors (Fig. 6e, f). The total IFNγ^+^ T cell displacements (measurement from point a to point b with a straight line) were the same for the two conditions (Supplementary Fig. 7G). These results suggest IFNγ^+^ T cells in combination-treated tumors were more actively surveilling their immediate environment compared to IFNγ^+^ T cells from control animals. The results align with a previous report showing IFNγ produced by cytotoxic T cells increases T cell motility and cytotoxicity^83^. Therefore, the T cell-intrinsic changes can contribute to an interferon licensing of the entire TME that may in turn lead to additional T cell infiltration into the tumor.

To interrogate the impact of IFNγ on the anti-tumor response induced by combination treatment, we performed an IFNγ blocking experiment. Adding a neutralizing IFNγ antibody to anti-PD-L1/TGFβ treatment completely inhibited the anti-tumor response caused by the combination, demonstrating the IFNγ dependency of the dual blockade (Fig. 6g). In addition, when we knocked out IFNGR1 in EMT6 tumor cells (Supplementary Fig. 7H), cells retained their ability to form tumors in vivo (Supplementary Fig. 7I), but the anti-tumor efficacy of the anti-PD-L1/TGFβ combination decreased (Fig. 6h). These data support the importance of the IFNγ response in inducing tumor rejection in combination treatment.

### Cross-compartmental interferon licensing is associated with improved overall survival of cancer patients

Due to the relationship between the increased number of T_SCL,_ which can fuel the T_EFF_ pool and TME interferon licensing, we hypothesized that an interferon-licensed TME might be predominantly found in inflamed human tumors and predictive of overall patient survival.

**Fig. 7:**
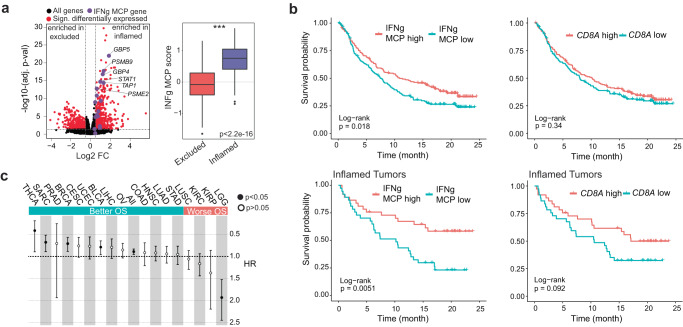
TME interferon licensing is associated with improved overall survival in human tumors. **a** (Left) Human immune-excluded (*n* = 134) versus inflamed (*n* = 74) tumor gene expression fold-changes (*x* axis) and their significance (y axis) (adj. *P* value from DESeq2). Human orthologues of IFNg MCP genes are highlighted in purple. (Right) IFNg MCP signature score between immune-excluded and inflamed tumor phenotypes. ***P < 2.2e-16. Wilcoxon rank-sum Test (two-sided). **b** (Top) Kaplan–Meier survival plot comparing survival probability (*y* axis) and follow-up time (*x* axis) for patients with locally advanced or metastatic urothelial carcinoma (IMvigor210; *n* = 348) receiving atezolizumab treatment. Groups were split by median: high (red, *n* = 174) or low (blue, *n* = 174) levels of IFNg MCP expression (left) or *CD8A* levels (right). (Bottom) Same plots as above, here showing only patients with inflamed tumor phenotype (high *n* = 37, low *n* = 37). **c** Forest plot depicting IFNg MCP signature overall survival hazard ratios (HRs; error bars represent 95% confidence interval) across specified TCGA indications (*n* = 7927). Solid circles represent *P* < 0.05 (Cox proportional hazards regression model). OS: Overall Survival. *P* values: 0.024 (THCA), 0.005 (SARC), 0.002 (BRCA), 0.018 (BLCA), 0.0008 (All), 5.09 e-08 (LGG). BLCA *n* = 408, BRCA *n* = 1085, CESC *n* = 303, COAD *n* = 444, HNSC *n* = 515, KIRC *n* = 515, KIRP *n* = 285, LGG *n* = 514, LIHC *n* = 368, LUAD *n* = 510, LUSC *n* = 487, OV *n* = 302, PRAD *n* = 494, SARC *n* = 255, STAD *n* = 412, THCA *n* = 501, UCEC *n* = 529. **a** Whiskers represent the minimum and maximum (unless points extend 1.5 * IQR from the hinge, then shown as individual points), the box represents the interquartile range, and the center line represents the median. THCA thyroid carcinoma, SARC sarcoma, PRAD prostate adenocarcinoma, BRCA breast invasive carcinoma, CESC cervical squamous cell carcinoma and endocervical adenocarcinoma, UCEC uterine corpus endometrial carcinoma, BLCA bladder urothelial carcinoma, LIHC liver hepatocellular carcinoma, OV Ovarian serous cystadenocarcinoma, COAD colon adenocarcinoma, HNSC head and neck squamous cell carcinoma, LUAD lung adenocarcinoma, STAD stomach adenocarcinoma, LUSC lung squamous cell carcinoma, KIRC kidney renal clear cell carcinoma, KIRP kidney renal papillary cell carcinoma, LGG brain lower-grade glioma.

To test the first hypothesis, we compared the expression profiles of human inflamed and excluded tumors from the IMVigor210 trial (348 patients with advanced-stage urothelial bladder cancer treated with atezolizumab (anti-PD-L1))^8^. Genes significantly upregulated in inflamed compared to excluded human tumors were enriched in human orthologues of our mouse IFNγ MCP signature. Similarly, the signature score was enriched in inflamed compared to excluded human tumors (Fig. 7a). Moreover, high levels of the IFNγ MCP predicted a favorable outcome, while the approximated amount of CD8 T cells, judged by *CD8A* expression, did not (Fig. 7b, top). Even when focusing on immune-inflamed tumors, we observed that high levels of IFNγ MCP predicted favorable survival, while this was not observed for *CD8A* alone (Fig. 7b, bottom). Furthermore, the association between high levels of the IFNγ MCP and favorable outcomes was found in a cross-TCGA analysis and within individual indications, such as sarcoma, breast cancer, or bladder cancer (Fig. 7c).

The significant association of IFNγ MCP with improved overall survival supports the notion that the main transcriptional change induced by the combination, a strong induction of interferon response across multiple cell types, is clinically associated with a better outcome.

## Discussion

We previously reported that TGFβ hampers response to anti-PD-L1 treatment in a CD8 T cell-dependent manner^8^. Here, we identified two key ways in which TGFβ influences CD8 T cells and consequently the entire TME: TGFβ impedes the expansion and differentiation of clonally diverse stem-like CD8 T_SCL_ cells and supports the maintenance of T_PEX_. At the same time, as T_SCL_ cells are the precursors of diverse T cell transcriptional states, including IFNγ^high^ CD8 T effector cells, TGFβ dampens the ability of CD8 T cells to trigger a TME-wide IFN licensing that is required for a complete response to the combination.

Our data are consistent with a model of two distinct populations of stem-like progenitor CD8 T cells^31^: we identified (1) a population similar to T stem/memory cells (TCF1/7^high^ PD-1^-/low^, clusters T1/7, that we refer to as T stem cell-like, T_SCL_) that is clonally diverse and (2) a population that expresses exhaustion markers, high TGFβ signaling pathway genes and shows high levels of TCR clonal expansion (T_PEX_, cluster T0, TCF1/7^+^ TOX^high^ PD-1^high^). Anti-PD-L1 and anti-TGFβ combination therapy leads to an increase of T_SCL_ and decreases T_PEX_ cells. TGFβ may be required for the activation and/or maintenance of the T_PEX_ CD8 T cell phenotype, in line with recent work that showed TGFβ signaling dependency of T_PEX_ in LCMV infection^40^. Additional experiments are needed to determine T_PEX_ cell fate with TGFβ blockade. Their decrease could be due to migration from the tumor, death, or de-differentiation into effector cells, as suggested by other studies^40^.

Furthermore, it remains to be experimentally confirmed if, in cancer, the increase in T_SCL_ is primarily due to in situ proliferation or if T_SCL_ are mainly derived from lymphoid organs. Our data suggest optimal anti-tumor efficacy is obtained when T cell trafficking between the lymph node and the tumor is intact, consistent with previous reports^84,85^. Further elucidating the relationship between CD8 T cells in the tumor and tumor-draining lymph nodes and their expression phenotypes and clonotypes will be essential to deepen our understanding of TGFβ in restraining their function in the anti-tumor response. In addition, while we show that in situ proliferation contributes to T_SCL_ expansion in combination, our analyses of other cell types in the TME revealed that combination treatment induces the expression of ligands in non-T cells that bind receptors specifically expressed by T_SCL_, such as *Cxcl9/10*. These computational analyses provide further insights into potential extrinsic mechanisms that can contribute to T_SCL_ expansion via cell-cell communications, especially with combination treatment. They further highlight the importance of interferon-mediated activation of chemokines and cytokines in non-T cells. Previous work in CAR T cells showed that successful CAR T cell killing requires intact interferon-gamma receptor signaling in tumor cells and downstream activation of adhesion pathways^86^. Further research will be needed to elucidate the importance of individual interferon-inducible pathways, chemokines, and cytokines in the context of anti-PD-L1 and anti-TGFβ blockade.

Still, our data demonstrate that CD8 T cells already present in the tumor can be sufficient to elicit curative anti-tumor immunity in a subset of animals, especially when combining PD-L1 and TGFβ blockade. TGFβ may limit the lifespan or function of the T_SCL_ compartment, allowing more cells to develop along the exhaustion trajectory rather than T effector differentiation, resulting in an accumulation of T_PEX_. While our data cannot answer this question definitively, it still provides some insights: when blocking new influx from the lymph node, combination therapy led to a better response compared to anti-PD-L1 alone, suggesting that in situ proliferation of CD8 T cells with stem-like phenotype may at least in part be driving TCR diversification, CD8 T effector cell rejuvenation, increase in IFNγ expression and tumor rejection.

While CD8 T cell expression phenotypes are not used for the stratification of clinical cohorts yet, tumors are histopathologically classified into inflamed and non-inflamed phenotypes using the abundance of intratumoral CD8 T cells as a critical criterion. Anti-PD-L1 and anti-TGFβ combination allowed the intratumoral migration of CD8 T cells, successfully converting the EMT6 immune-excluded phenotype into an inflamed tumor. Our analysis suggests that the T cell landscape and the entire TME, including myeloid, tumor, and fibroblastic cells, are remodeled upon successful checkpoint blockade. The greater migratory capacity of IFNγ^hi^ T effector cells observed following combination treatment could further increase the amount of IFNγ sensed by cells of the various cell compartments as previously shown^87,88^. We observed cross-compartmental IFN licensing upon TGFβ/PD-L1 dual blockade. All major populations in the TME increased expression of antigen presentation-related genes and T cell stimulatory cytokines and chemokines. These findings suggest that TGFβ may dampen CD8 T cell activity by restraining their provision of IFNγ and that success of combination therapy depends on ensuing IFNγ signaling in multiple non-T cell compartments of the tumor ecosystem. In line with this hypothesis, IFNγ is required for successful tumor rejection by dual blockade of TGFβ and PD-L1, and knocking out IFNγ receptor in tumor cells leads to decreased complete response to dual PD-L1 TGFβ blockade. Our observations align with previous work on cross-talk between IFN and TGFβ signaling pathways^89–93^. While previous studies have described an association between IFNγ-related genes and response to checkpoint inhibition, the gene sets developed in these studies contained a mixture of interferon response genes and genes expressed by CD8 T cells, such as *IFNG*^5–7^. Our study focused on identifying a gene signature induced in non-T cells mediated by IFNγ. Using this signature, we can provide evidence of a direct clinical association between interferon licensing, likely across multiple compartments of the TME, and overall patient survival.

The data presented here are improving our understanding of the pharmacodynamic effects of TGFβ blockade in combination with immune checkpoint inhibitors that may need to be considered for future clinical trial design.

## Methods

Our research complies with all relevant ethical regulations. All animal activities in the research studies presented here were conducted under protocols approved by the Genentech Institutional Animal Care and Use Committee (IACUC).

### Cell lines

The EMT6 murine mammary carcinoma cell line was obtained from American Type Culture Collection (ATCC; Manassas, VA). EMT6 cells were engineered to express either THY1.1 (for flow cytometry and RNA-seq experiments) or mAPPLE (for in vivo imaging).

To generate an EMT6-Thy1.1 cell line, a pLVX-puromycin backbone vector containing murine Thy1.1 was packaged into viral particles by transfecting HEK293T cells with Lenti-X packaging single shots (Clontech, Mountainview, CA). After 48 h, the virus-containing media was collected, filtered, and concentrated with a Lenti-X concentrator (Clontech). EMT6 cells were then transduced with the virus and polybrene (8 µg/mL) with 2 h spin at 30 °C and 2000 rpm. Cells were then placed under puromycin selection and expanded for 1 week. To obtain a stable Thy1.1 high cell line, the top 10% of Thy1.1+ cells were sorted as single cells, and individual clones were expanded. After expansion, clones were screened for high Thy1.1 expression that was maintained after 2–3 weeks in culture, and four clones were chosen. Finally, two of these lines were injected s.c. into mice, and the tumors were collected after about 2 weeks to confirm that Thy1.1 expression was maintained in vivo. Clone 5 was used for in vivo experiments.

To generate EMT6-mAPPLE, 1 × 10^6^ EMT6 cells were seeded per 10-cm cell culture dish in RPMI-medium. After 24 h, EMT6 cells were transfected using LipofectamineTM 3000 Reagent (Thermo Fisher Scientific, Waltham, MA) according to the manufacturer’s instruction, 4 µg plasmid DNA together with 8 µg of piggyBacVector DNA were used per dish. After expansion, cells were single-cell sorted based on mApple-high expression, and clone E1 was selected for further in vivo use based on mApple expression.

EMT6 IFNGR1 KO cells were generated at Synthego (Redwood City, CA) using CRISPR KO (Guide RNA sequence: UGGUAUUCCCAGCAUACGAC; Guide RNA cut location chr10:19,597,466; Exon targeted: 2). The loss of IFNγ response in the EMT6 IFNGR1 KO cells was validated in vitro by stimulating the cells with mouse recombinant IFNγ (catalog number n 485-MI, R&D Systems, Minneapolis, MN) for 30 min at 37 °C and by analyzing STAT1 phosphorylation by flow cytometry.

### Mice

Female BALB/c mice were obtained from Charles River Laboratories (Hollister, CA). Female IFNγ-YFP reporter mice, aka GREAT mice: the “interferon-gamma reporter with endogenous polyA transcript” (GREAT) allele has an IRES-eYFP reporter cassette inserted between the translational stop codon and 3’ UTR/polyA tail of the interferon-gamma (IFNγ) gene. The bicistronic IFNγ-IRES-eYFP mRNA is under control of the endogenous IFNγ promoter/enhancer regions with proper regulation defined by the endogenous 3’ UTR and polyA tail. This allows the translation of both IFNγ and eYFP from the same mRNA and therefore the analysis of IFNγ–competent cells in vivo by detection of YFP expression without the need for restimulation. The IFNγ-YFP reporter mice were licensed from UCSF and backcrossed to the BALB/c background.

All mice were housed at Genentech in individually ventilated cages within animal rooms maintained on a 14:10-h, light:dark cycle. Animal rooms were temperature and humidity-controlled, between 68 and 79 °F and 30 and 70%, respectively, with 10 to 15 room air exchanges per hour. Mice were acclimated to study conditions for at least 3 days before tumor cell implantation. Animals were 8–10 weeks old. Only animals that appeared to be healthy and free of obvious abnormalities were used for the studies.

Animals were maintained in accordance with the *Guide for the Care and Use of Laboratory Animals*. Genentech is an AAALAC-accredited facility, and all animal activities in the research studies were conducted under protocols approved by the Genentech IACUC.

### In vivo studies

EMT6 cells were cultured in Roswell Park Memorial Institute (RPMI) 1640 medium plus 2 mM l-glutamine with 10% fetal bovine serum (FBS; HyClone, Waltham, MA). Cells in log‑phase growth were centrifuged, washed once with Hank’s balanced salt solution (HBSS), counted, and resuspended in 50% HBSS and 50% Matrigel (BD Biosciences; San Jose, CA) at a concentration of 1 × 10^6^ cells/mL for injection into mice. Mice were inoculated with 1 × 10^5^ cells in 100 μL of HBSS: Matrigel (1:1). EMT6 cells were inoculated in the left mammary fat pad #5 of the mouse. When the tumor reached a volume of 130–230 mm^3^, animals were distributed into treatment groups based on tumor volume and treated with isotype control antibodies (mouse IgG1 anti-gp120, 20 mg/kg first dose followed by 15 mg/kg thereafter), anti-PD-L1 (mouse IgG1 clone 6E11, 10 mg/kg first dose followed by 5 mg/kg thereafter), anti-TGFβ (mouse IgG1, 10 mg/kg), or a combination of anti-PD-L1 with anti-TGFβ antibody (Supplementary Table 4). Antibodies were administered three times a week for 21 days, the first dose intravenously, and subsequent doses intraperitoneally.

For FTY720 experiments, FTY720 oral administration started at the same time as the antibody treatments. FTY720 1 mg/kg was administered by oral gavage daily for 21 days. For efficacy studies, tumors were measured two times per week by caliper.

Mice were euthanized immediately if tumor volume exceeded 2000 mm^3^ (maximal tumor volume permitted by the Genentech IACUC), or if tumors ever fall outside the IACUC Guidelines for Tumors in Rodents. Mice were considered complete responders (CR) when their tumors became smaller than 32 mm^3^ (limit of detection) and remained undetectable. Efficacy studies were terminated about 40–50 days after initiation of treatment. No mice met the criteria for euthanasia because of body weight loss nor exhibited adverse clinical signs during the studies.

For IFNγ neutralizing experiments, an anti-mouse IFNγ antibody (InVivoPlus Clone XMG1.2, Catalog number BP0055; BioXcell, Lebanon, NH) was administered at 12.5 mg/kg at the same time as the other antibodies (three times a week for 21 days, first dose intravenously, subsequent doses intraperitoneally).

For EdU pulse experiments, 200 μl of 5 mg/ml EdU (5-ethynyl-2′-deoxyuridine, Component A, Life Technologies, cat. no. A10187) were injected intraperitoneally 24 h before mouse euthanasia.

For IHC, flow cytometry analysis, proteomic analysis or FACS sorting, and subsequent RNA-seq analysis, mice were euthanized 7 days after treatment initiation, and tumors were collected.

#### Tumor growth analysis

Analyses and comparisons of tumor growth were performed using a package of customized functions in R (Version 4.1.0 (2021-05-18); R Foundation for Statistical Computing; Vienna, Austria), which integrates software from open-source packages (e.g., lme4, mgcv, gamm4, multcomp, settings, and plyr) and several packages from tidyverse (e.g., magrittr, dplyr, tidyr, and ggplot2)^94^. Briefly, as tumors generally exhibit exponential growth, tumor volumes were subjected to natural log transformations before analysis. All raw tumor volume measurements from 0 to 8 mm^3^ were judged to reflect complete tumor absence and were converted to 8 mm^3^ before natural log transformation. A generalized additive mixed model was then applied to describe the changes in transformed tumor volumes over time using regression splines with automatically generated spline bases. This approach addresses both repeated measurements from the same study subjects and moderate dropouts before the end of the study.

### Intravital two-photon imaging of YFP+ cells in dorsal skinfold chamber

The surgical procedure for installation of the dorsal skinfold chambers (DSFC) into IFNγ-YFP reporter mice was adapted from previous work^95^. Briefly: after anesthesia, the chamber was sutured into the skinfold with care to ensure that vascular integrity was maintained. Following surgery, the mouse was transferred immediately to a heated cage for recovery. Once ambulatory, mice were housed singly with food and water on the cage floor.

Each mouse was then viewed under a dissecting stereomicroscope to confirm blood circulation within the chamber window. 48 h post DSFC installation, 750k EMT6-mApple tumor cells were implanted inside the DSFC chamber. Tumor growth was monitored by caliper measurements every 2–3 days and once palpable tumors reached 150–200 mm^3^ size (day 10 post-implant) mice were distributed into treatment groups and treated with isotype control antibodies (mouse IgG1 anti-gp120, 20 mg/kg first dose followed by 15 mg/kg thereafter) and anti-PD-L1 (mouse IgG1 clone 6E11, 10 mg/kg first dose followed by 5 mg/kg thereafter) in combination with anti–PAN-TGFβ (mouse IgG1, 10 mg/kg). Treatment was administered every 2–3 days for 1 week.

Live imaging of YFP+ cells within tumors was conducted routinely beginning on day 10 post-tumor implantation and ending on day 24 (day 14 post-treatment). Mice were imaged on a two-photon laser-scanning microscope (Ultima In Vivo Multiphoton Microscopy System; Prairie Technologies) using two Ti:sapphire lasers (MaiTai DeepSee Spectra Physics; Newport) tuned to 910 nm and 980 nm with a 16x (numerical aperture 0.8) objective lens (Nikon) to allow 4-color detection: Ch1 (far-red, vascular dye), Ch2 (red, mApple tumor cells), Ch3 (green, YFP+ cells) and Ch4 (blue, collagen). Mice were placed on a custom scope stage designed to maintain mouse body temp at 37 °C and provide anesthesia. A built-in spring-loaded C-clamp was gently placed on top of the chamber to reduce breathing artifacts while imaging. GenTeal eye lubricant (Alcon) was applied to the DSFC window to allow proper immersion of the 16x objective. Mice were injected IV with 100 µl AngioSense EX (Perkin Elmer) to label vasculature. Epifluorescence illumination (X-Cite Series 120Q unit, Lumen Dynamics) was used to locate mApple+ EMT6 tumor and select for 2-photon imaging. Second harmonic generation (SHG) was used to locate the tumor periphery via collagen deposition. Z-stacks were set using the SHG signal to guide upper and lower limits. The total image stack depth did not exceed 100 µm. Time-lapse Z-stacks were collected at 512 × 512 resolution, 2.8 Us dwell-time, and 1.5-micron z-step with a total stack time of ~45 s. Total recording time ranged from 10–45 min per mouse. Fluorescence signals from YFP+ cells, mApple+ tumor cells, vasculature (AngioSense), and collagen (SHG) were collected with the following filters: green (BP 525/30), red (BP 618/570), far-red (692LP), and blue (BP 452/45). Imaging was repeated at three other random regions of the tumor up for a maximum imaging duration of 2 h. When the imaging session was completed, mice were allowed to recover on a heating pad and once ambulatory it was returned to its cage.

For image analysis, ImageJ/Fiji, Matlab (Mathworks), and Imaris (Bitplane) were used. YFP+ cells dynamics were quantified using Imaris (Spot and surface detection) and Matlab routines. Statistical analysis was performed using GraphPad Prism v7.02. Data are expressed as mean, mean +/− SEM. For the comparison of the two groups, a two-way ANOVA was used. A level of *P* < 0.05 was considered statistically significant.

### Immunohistochemistry on mouse tissue

Tumors were collected 7 days after treatment initiation. Tumors were fixed in 10% neutral buffered formalin (NBF) and paraffin-embedded. IHC was performed on 4-μm thick paraffin-embedded tissue sections mounted on Superfrost Plus glass slides. Staining was performed on the Lab Vision Autostainer (Thermo Fisher Scientific, Waltham, MA). Sections were de-paraffinized and rehydrated to deionized water. Antigen retrieval was performed with 1× DAKO Target Retrieval Solution (Agilent Technologies, Carpinteria, CA) for 20 min at 99 °C and cooled to 74 °C. Subsequently, endogenous peroxidase was quenched by incubating in sections in 3% H_2_O_2_ for 4 min at room temperature. Slides were then incubated with hamster anti-mouse CD8a (clone 1.21E3.1.3, Genentech, Inc.) at 5 µg/ml for 60 min at room temperature followed by biotinylated goat anti-hamster IgG (Vectorlabs; Burlingame, CA), detected with Vectastain ABC-HRP (Vectorlabs; Burlingame, CA) and visualized with Pierce metal-enhanced DAB (Thermo Scientific; Fremont, CA). For Masson trichrome counterstaining, the sections were fixed in Bouin’s fixative (American MasterTech, Lodi, CA; FXBOUGAL) at 60 °C for 1 h then rinsed with tap water and stained with Weigert’s hematoxylin with dH2O and 95% ethanol for 5 min. Following wash with tap water for 5 min, rinse with dH2O, slides were stained with Biebrich scarlet-acid fuchsin (Spectrum Chem, Vernon Hills, IL; CAS 3761-53-3, Spectrum Chem, Vernon Hills, IL; CAS 3244-88-0, American MasterTech, Lodi, CA; AHORG25) (1:100 vol/vol) in 0.2% acetic acid (VWR, Radnor, PA;BDH3092) at 60 °C for 30 s. Slides were then serially incubated in 0.2% acetic acid (VWR, Radnor, PA;BDH3092) in dH2O for 10 min, 5.0% phosphotungstic acid (American MasterTech, Lodi, CA;AHPPTA125) in dH2O for 20 min, 0.2% acetic acid (VWR, Radnor, PA;BDH3092) in dH2O for 2 min and stained for collagen using 3.0% aniline blue (American MasterTech, Lodi, CA; AHABL25) in dH2O (3:1 vol/vol) for 1 min. Slides were then decolorized and coverslipped.

#### CD8 T cell infiltration analysis

Bright-field images were acquired by Hamamatsu Nanozoomer automated slide-scanning platforms at a final magnification of Å~200. The images were analyzed with the 2016b version of the Matlab software package (MathWorks). Regions of interest (ROIs) were defined by a pathologist. Cells marked with CD8+ that lay within the ROI border were identified by intensity thresholding and simple morphological filtering. Immune cell infiltration was evaluated for each slide (i.e., each mouse) by calculating the nearest distance to the ROI border over all CD8+ marked cells within the ROI on that slide. Distances were then normalized per slide by dividing by the maximum distance that any CD8+ cell could potentially travel from the ROI border. Averages of normalized distances were then calculated to arrive at normalized mean distances. Normalized mean distances were then pooled across the three studies and analyzed by linear regression with the treatment group as a fixed categorical variable and ROI area and the total number of CD8+ cells within the ROI as covariates. Covariate-adjusted means and 95% confidence intervals were reported for each treatment group. Pairwise comparisons among treatment groups were made using Tukey’s HSD. All analyses were performed using R (R Foundation for Statistical Computing; Vienna, Austria), and distance calculations were made using the R package “spatstat”^96^.

### Preparation of single-cell suspension and antibody staining for flow cytometry and sorting

Tumors were collected 7 days after treatment initiation. Tumors were weighed and enzymatically digested using a cocktail of dispase, collagenase P, and DNaseI for 45 min at 37 °C, to obtain a single-cell suspension. Cells were counted using a Vi-CELL XR (Beckman Coulter, Brea, CA).

For flow cytometry analysis, cells were first incubated with mouse BD Fc block (5 µg/ml) and LIVE/DEAD® Aqua Fixable Dead Cell Stain for 30 min on ice. The cells were then stained with the following antibodies. For the T cells staining: CD45 BV605 (0.67 µg/ml), TCR-β PE or APC (2 µg/ml), CD8 APC-Cy7 (1 μg/ml), LAG3 BV785 or Percp-Cy5.5 (2 μg/ml), TIM3 PE-Cy7(2 μg/ml), PD-1 BV711 (1 μg/ml), CD39 PE (2 μg/ml) for 30 min on ice. Cells were fixed and permeabilized eBioscience™ Foxp3/Transcription Factor Staining Buffer Set to stain for Granzyme B FITC (5 μg/mL), IFNγ PE-Cy7(2 μg/ml), and TCF1 AF647 (1:50). For E22 tetramer and EdU staining, cells were first stained with E22 Tetramer or LCMV tetramer PE (1:12.5 dilution) for 20 min at room temperature. Then cells were stained with the following antibodies: CD45 BV605 (0.67 µg/ml), LAG3 APC (2 µg/ml), CD8 APC-Cy7 (1 μg/ml), CD90.2 BV785 (1 µg/ml), TIM3 PE-Cy7(2 μg/ml), PD-1 BV711(1 μg/ml). After surface staining EdU staining was performed using the CLICK-IT EDU PLUS Alexa Fluor 488 KIT following manufacture protocol (Thermo Fisher Scientific). Flow Cytometry data were collected with a BD LSRFortessa cell analyzer or FACSymphony (BD Biosciences, San Jose, CA) using BDFACSDiva software (versions 8.0.1 and 9.1, BD Biosciences, San Jose, CA). Flow data were analyzed in FlowJo, and exported to CSV files and statistical analysis was performed using the R package tidyverse, dplyr, and FSA. Treatment-induced changes were identified with Kruskal–Wallis ANOVA followed by Dunn’s test with multiple testing correction via the Benjamini–Hochberg method.

For sorting (scRNA-seq and bulk RNA-seq of TME populations), cells were first enriched for live cells using Dead Cell Removal Kit following the manufacturer’s instructions. Cells were then incubated with mouse BD Fc block (5 µg/ml) for 30 min on ice. And then stained for 30 min on ice with the following antibodies: CD45 PE-Cy7 (1 µg/ml), TCR-β APC (1 µg/ml), THY1.1 AF488 (2.5 µg/ml), PDPN PE (0.5 µg/ml), CD31 BUV737 (1 μg/ml), CD11b BUV395 (1 μg/ml), plus one of the following Hashtag antibodies (one for each replicate, 5 µg/ml): TotalSeq™-B0301 (barcode sequence: ACCCACCAGTAAGAC), -B0302 (barcode sequence: GGTCGAGAGCATTCA), -B0303 (barcode sequence: CTTGCCGCATGTCAT), -B0304 (barcode sequence: AAAGCATTCTTCACG), -B0305 (barcode sequence: CTTTGTCTTTGTGAG). Just before sorting cells from the same treatment group were pulled together and stained with 7AAD and eBioscience™ Calcein Blue AM Viability Dye. Four population were sorted: T cell (7AAD- Calcein blue+ CD45 + THY1.1- TCRb + ), fibroblasts (7AAD- Calcein Blue+ CD45- THY1.1- CD31- PDPN + ), myeloid cells (7AAD- Calcein Blue+ CD45 + THY1.1- CD11b + ) and tumor cells (7AAD- Calcein Blue+ CD45- THY1.1 + ). Cells were sorted using a BD FACSAria™ Fusion flow cytometer (BD Biosciences, San Jose, CA). For scRNA-seq, 50k cells for each population were sorted in 300 UL of MACS buffer kept at 4 °C. Cells were then counted and resuspended at an adequate concentration for loading into the 10X chips. For bulk RNA-seq, 5k cells for each population per mouse were sorted in 100 UL of RA1 buffer + 2 UL of TCEP (NucleoSpin RNA XS) spun down, and immediately frozen in dry ice. Samples were conserved at −80 °C until RNA extraction.

For sorting (TCR/scRNA-seq), cells from tumor digestion were first enriched for live cells using Dead Cell Removal Kit following the manufacturer’s instructions. Cells were then incubated with mouse BD Fc block (5 µg/ml) for 30 min on ice. The cells were then stained for 30 min on ice with the following antibodies: CD45 BUV395 (1 µg/ml), CD11b BV650 (0.5 µg/ml), CD19 BV650 (1 µg/ml), NK1.1 BV650 (1 µg/ml), CD90.2 BV785 (1 µg/ml), CD44 FITC (1 µg/ml), plus one of the following Hashtag antibodies (one for each replicate, 5 µg/ml): TotalSeq™-C0301 (barcode sequence: ACCCACCAGTAAGAC), -C0302 (barcode sequence: GGTCGAGAGCATTCA), -C0303 (barcode sequence: CTTGCCGCATGTCAT), -C0304 (barcode sequence: AAAGCATTCTTCACG), -C0305 (barcode sequence: CTTTGTCTTTGTGAG), -C0306 (barcode sequence: TATGCTGCCACGGTA). Just before sorting cells from the same treatment group/ tissue of origin were pulled together and stained with 7AAD and eBioscience™ Calcein Blue AM Viability Dye. T cells from the tumor were sorted as 7AAD-CB + CD45 + CD11b-CD19-NK1.1-CD90 +. Cells were sorted using a BD FACSAria™ Fusion flow cytometer (BD Biosciences, San Jose, CA). For TCR/scRNA-seq, 75k cells T cells were sorted in 300 UL of MACS buffer kept at 4 °C. Cells were then counted and resuspended at an adequate concentration for loading into the 10X chip.

For E22-specific CD8 T cell sorting (TCR/scRNA-seq), cells from tumor digestion were first enriched for live cells using Dead Cell Removal Kit following the manufacturer’s instructions. Cells were then incubated with mouse BD Fc block (5 µg/ml) for 30 min on ice. Cells were stained with E22 Tetramer PE (1:12.5 dilution) for 20 min at room temperature. Cells were then stained for 30 min on ice with the following antibodies: CD45 BUV395 (1 µg/ml), CD11b BV650 (0.5 µg/ml), CD19 BV650 (1 µg/ml), NK1.1 BV650 (1 µg/ml), CD90.2 BV785 (1 µg/ml), CD8 APC-Cy7 (1 μg/ml), CD4 BV711 (1 µg/ml) plus one of the following Hashtag antibodies (one for each replicate, 5 µg/ml): TotalSeq™-C0301 (barcode sequence: ACCCACCAGTAAGAC), -C0302 (barcode sequence: GGTCGAGAGCATTCA), -C0303 (barcode sequence: CTTGCCGCATGTCAT), -C0304 (barcode sequence: AAAGCATTCTTCACG). Just before sorting cells from the same treatment group/ tissue of origin were pulled together and stained with 7AAD and eBioscience™ Calcein Blue AM Viability Dye. T cells from the tumor were sorted as 7AAD-CB + CD45 + CD11b-CD19-NK1.1-CD90 + CD8 + E22 +. Cells were sorted using a BD FACSAria™ Fusion flow cytometer (BD Biosciences, San Jose, CA). For TCR/scRNA-seq, 16 to 50k cells T cells were sorted in 300 UL of MACS buffer kept at 4 °C. Cells were then counted and resuspended at an adequate concentration for loading into the 10X chip.

### scRNA-seq and TCR-seq sample processing

After FACS sorting, cells were processed using the Chromium Single Cell Gene Expression 3′ Library and Gel Bead kit following the manufacturer’s instructions (10x Genomics). Cells were counted and checked for viability using a Vi-CELL XR cell counter (Beckman Coulter) and then injected into microfluidic chips to form Gel Beads-in-Emulsion (GEMs) in the 10x Chromium instrument. Reverse transcription was performed on the GEMs, and reverse-transcribed products were purified and amplified. Gene expression libraries and hashtag libraries were made from the cDNA, profiled using a Bioanalyzer High Sensitivity DNA kit (Agilent Technologies), and quantified with a Kapa Library Quantification kit (Kapa Biosystems). HiSeq 4000 (Illumina) was used to sequence the libraries.

For TCR-sequencing, the cells were processed using the Chromium Single Cell Immune Profiling 5’ v2 kit (10x Genomics). After reverse transcription and cDNA amplification, TCR enrichment was performed using mouse T cell primer mixes according to 10x’s recommendations. Subsequently, libraries were constructed for gene expression, TCR, and hashtags. Libraries were profiled with the Bioanalyzer High Sensitivity DNA (Agilent Technologies) and quantified with a Kapa Library Quantification kit (Kapa Biosystems). HiSeq 4000 (Illumina) was used to sequence the libraries.

### (sc)RNA-seq data analyses

#### Bulk RNA-seq of human IMVigor210 and TCGA data

Whole-transcriptome data from patients enrolled in the anti-PD-L1 (atezolizumab) immunotherapy trial IMvigor210 (NCT02951767, NCT02108652;^8,97^), were generated as described previously^98^, normalized to counts per million (CPM) and log2 transformed. IFN response signature scores were calculated as the median value of human orthologs of the MCP in each sample after z-score transformation of the expression of each gene. Batch-corrected normalized TCGA Pan-Cancer mRNA data were obtained from UCSC Xenabrowser (https://xenabrowser.net/) (*N*  =  11,060). Samples containing NA expression values were removed. We additionally filtered the data to only contain samples from primary solid tumors (sample code 01; *N*  =  9702). Survival data were obtained from Supplementary Table 1^99^ and linked to the Pan-Cancer dataset using the unique TCGA participant barcode. Indications with fewer than 200 patients were excluded from the analysis (final dataset: *N*  =  7927 patients). IFN response scores were calculated as described for the IMVigor210 trial. Association with survival across TCGA data was determined with multivariate Cox regression and TCGA indication as a covariate, as well as univariate Cox regression analysis within each indication.

#### Bulk RNA-seq of mouse data

RNA was isolated using NucleoSpin RNA XS isolation kit (TAKARA Bio USA Inc.) following the manufacturer’s instruction. RNA was eluted in 10 UL of RNAse-free water. Total RNA was quantified with Qubit RNA HS Assay Kit (Thermo Fisher Scientific), and quality was assessed using RNA ScreenTape on TapeStation 4200 (Agilent Technologies). cDNA library was generated from 2 ng of total RNA using Smart-Seq V4 Ultra Low Input RNA Kit (Takara). In total, 150 picograms of cDNA were used to make sequencing libraries by Nextera XT DNA Sample Preparation Kit (Illumina). Libraries were quantified with Qubit dsDNA HS Assay Kit (Thermo Fisher Scientific), and the average library size was determined using D1000 ScreenTape on TapeStation 4200 (Agilent Technologies). Libraries were pooled and sequenced on HiSeq 4000 (Illumina) to generate 30 millions single-end 50-base pair reads for each sample. RNA-seq data were analyzed using HTSeqGenie (https://bioconductor.org/packages/release/bioc/html/HTSeqGenie.html) as follows: first, reads with low nucleotide qualities (70% of bases with quality <23) or matches to ribosomal RNA and adapter sequences were removed. The remaining reads were aligned to the mouse reference genome (mm10) using GSNAP, allowing a maximum of 2 mismatches per 75-base sequence. Subsequently, the number of fragments was calculated by counting the number of uniquely mapped concordant pairs to the exons of each RefSeq gene using the *summarizeOverlaps* function from the GenomicAlignments package^100^. Expression levels of each gene were normalized to Reads Per Kilobase of transcript, per Million mapped reads (RPKM) and log2 transformed. Heatmaps of gene expression were generated on gene-wise z-scored expression values using the pheatmap package (https://cran.r-project.org/web/packages/pheatmap/index.htm). Principal component analysis was performed on the 2000 most variable genes (genes with the highest interquartile range) robustly expressed by CD8 T cells in the single-cell dataset. Scores for gene signatures identified in scRNA-seq data (20 most strongly upregulated genes in a cluster, judged by logFC) were calculated as the z-scored average expression of the marker genes (z-score of average (Log2(RPKM + 1))).

#### ScRNA-seq of mouse data

Single-cell RNA-seq data for each library from each cell type was processed with CellRanger *count* (10x Genomics) using a custom reference gene annotation based on mouse reference genome GRCm38/mm10 and GENCODE gene models. UMI count tables were read in R with the Seurat^101^ package and counts were normalized to (CPM/100 + 1) and log transformed. UMI counts of barcode antibodies to label individual replicates were first normalized with centered log ratio (CLR) transformation per cell (Seurat, *NormalizeData*), followed by antibody assignment where the top 95% quantile hashtags were considered a positive signal (*HTODemux*). Individual libraries were merged into a Seurat object. Cells with less than 300 measured genes or over 5% mitochondrial counts were removed. Only cells labeled as “singlets” based on HTO counts were included. The top 2000 most variable genes were selected via variance stabilizing transformation (vst) (*FindVariableFeatures*) and their expression was scaled (*ScaleData*). Principal component analysis was then performed in this gene space (*RunPCA*). Clustering was carried out based on the shared nearest neighbor between cells (*FindNeighbors*) and graph-based clustering (*FindClusters*). For graph-based clustering and generation of a UMAP reduction (*RunUMAP*) the same number of principal components was used as input^101^.

For 25,063 cells in the T cell sort, 30 PCs and a resolution of 0.5 were used to identify clusters. On the entire T cell object, clusters were annotated as either Tregs (*Cd3d*+ *Foxp3*+), conventional CD4 T cells (*Cd3d*+*Cd4*+ *Cd40lg*+), CD8 T cells (*Cd3d*+*Cd8*+) and naive T cells (*Cd3d*+ *Sell*+). Data from CD8 T cells was re-processed in Seurat as described above using a resolution of 0.6 and 22 PCs.

For 37,773 cells in the fibroblast sort, 30 PCs and a resolution of 0.05 were used to identify clusters. On the entire fibroblast object, clusters were annotated as either fibroblast (*Lum*+, *Dcn*+, *Pdgfra*+), epithelial cells (*Epcam*+, *Krt19*+), immune cells (Tyrobp+, *Ptrpc*+) and EMT tumor cells (*Krt8*+ *Msln*+, *Hmga2*+). Data from *Dcn*+, *Pdgfra*+ fibroblasts was re-processed in Seurat as described above using the following parameters: 25 PCs, resolution: 0.1.

For 45,024 cells in the tumor sort, 20 PCs and a resolution of 0.3 were used to identify clusters. Due to a large number of proliferating tumor cells, cell cycle was regressed out to avoid a separation of tumor cells by cell cycle stages. For this regression, cells were first scored for cell cycle phase markers using the *CellCycleScoring* function in Seurat. Subsequently, these scores were provided to the scaling function before PCA (*vars.to.regress* argument in the *ScaleData* function, *S.Score* and *G2M.Score* provided).

For 26,867 cells in the myeloid sort, 30 PCs and a resolution of 0.3 were used to identify clusters. On the entire myeloid object, clusters were annotated as either NK cells (*Ncr1*+, *Cd8-*) or non-NK myeloid cells. Data from non-NK myeloid cells was re-processed in Seurat as described above using the following parameters: 27 PCs, resolution: 0.1.

Markers for each cluster were detected using the *FindAllMarkers* function in Seurat with default parameters. The average expression of markers was calculated for each cluster using the *AverageExpression* function in Seurat, and per-gene z-scores were calculated for visualization with the pheatmap package. Density plots of cells were generated using the UMAP coordinates of cells from each condition using the LSD R package (https://cran.r-project.org/web/packages/LSD/index.html).

Cluster population frequency changes between conditions were evaluated with Dunn’s test with *P* values adjusted via Benjamini–Hochberg following an ANOVA using speckle (for clusters with *P* < 0.05, “asin” transformation, https://github.com/Oshlack/speckle). Signatures scores for each gene sets of interest were calculated by taking the average expression of the gene set subtracted from the average expression of a control set (*AddModuleScore*). Gene set enrichment analysis was performed with the package fgsea^102^ via *fgseaMultilevel*. Trajectory analysis of CD8 T cells was performed with slingshot^103^, where the top ten principal components previously calculated in Seurat were used as an input.

To identify differential gene expression comparing any two treatments, all reads from cells of the same replicate were first pooled into a pseudobulk sample. Replicates were then grouped in a “pseudo bulk” expression matrix and pairwise comparisons between conditions were carried out with DESeq2^104^ taking replicate information into account. Enriched pathways for up and downregulated genes were calculated with the *enrichr* package^105^, using databases including BioCarta, GO molecular function, WikiPathways Mouse, MSigDB Hallmark and Oncogenic Signatures, and Panther from enrichR^105^. Pathways with less than five overlapping genes between the query and pathway genes were removed.

To identify multicellular programs (MCP), consensus non-negative matrix factorization (cNMF) was applied to cells from each of the three cell types separately^106^. The number of components k was determined based on stability. For tumor cells and fibroblasts, we used *k* = 17, for myeloid cells *k* = 19. Genes were then ranked by their contribution to each program and the top 50 genes were used for pathway enrichment analysis as described before. The cluster T1 signature was calculated by passing the 20 genes with the highest log2 fold change to *AddModuleScore* from Seurat^101^.

Ligand-receptor interactions were identified by using the ligand-receptor interaction databased from the nichenetr R package^107^ and filtered for interactions from the KEGG database. We additionally filtered the database for interactions that involved receptors enriched in T_SCL_ compared to other T cells in our dataset (p_val_adj <1e-03 & percent expressing T_SCL_ > 35). Last, we pruned interactions to those involving ligands that were expressed in at least 10% of sender cells (tumor, myeloid or fibroblasts separately). The average expression of the remaining ligand-receptor pairs was visualized as heatmaps of average expression in each condition (ligand) and the fold change compared to non-T_SCL_ T cells (receptors).

#### RNA velocity analysis

Fastqs were mapped with kallisto bustools^108^ to an index including intronic sequences obtained using BUSpaRse (2021) with the lengths of reads designated, 90 nt for scRNA/TCR-seq data and 98 nt for scRNA-seq. Spliced and unspliced counts for CD8 T cells were collated using Seurat and imported into scVelo^109^. Genes with fewer than 20 spliced and unspliced counts combined were removed. Counts were normalized by cell, and the top 2000 variable genes were extracted and log normalized (*scvelo.pp.filter_and_normalize*). First- and second-order moments were calculated using embeddings from the canonically processed data seen in Figs. 2 and 3 with 30 PCs and 30 neighbors (*scvelo.pp.moments*). The splicing kinetics were calculated for all highly variable genes (*scvelo.tl.recover_dynamics*) and used to estimate dynamic models of splicing kinetics (*scvelo.tl.velocity*). First order moments of spliced and unspliced counts per cell, the steady-state ratio, and the dynamic model were then plotted for *Ifng* (*scvelo.pl.velocity*).

#### ScTCR-seq

Single-cell RNA-seq data for each T cell library was pre-processed as described above. Barcode antibodies for cell hashing were demultiplexed and assigned by a custom R wrapper of the DemuxEM package^110^. Individual libraries were merged into a Seurat object. Only cells designated as “singlet” were retained. T cell receptor genes were removed to avoid clonal-based clustering. Dimensionality reduction and clustering were performed as described above, here using the following parameters: 30 PCs and a resolution of 0.8, followed by removal of contaminants expressing *Ncr1*, *Dcn*, *Siglech*, *Cd74*, *Xist*, and *Tcrg*, and additional filtering of remnant NK cells expressing *Ncr1* at resolution 0.8, followed by rescaling and reclustering as described above. CD8 T cell clusters were identified as those expressing *Cd8a* with low levels of *Cd4*, *Foxp3*, *Lef1*, and *Ncr1* and extracted. The finalized CD8 T cell object was rescaled and clustered at resolution 0.8 followed by the removal of naive cells expressing *Lef1*, *Tcf7*, and *Sell* as well as cells expressing *Ncr1* and *Cd4*. The finalized CD8 T cell object was rescaled and clustered at resolution 0.6. Marker genes and cluster population change were calculated as mentioned above. To determine the similarities of the CD8 T clusters between two separate experiments, scRNA or scTCR, ClusterMap^111^ was used to perform cluster marker comparison (*cluster_map*).

ScTCR-seq data was processed with CellRanger *vdj* to generate contig count tables for each sequencing library. TCR contig tables were then split by individual mice based on cell hashing assignments. Contigs were next assigned to their respective cell in the Seurat expression object based on the cellular barcode information. A modified version of the *combineTCR* function from scRepertoire^112^ was used to assign between 0 to 2 alpha and 0 to 2 beta chains per cell ordered by the most reads. Clonal assignments were made using the nucleotide sequence (CTnt). TCR diversity analysis was performed with scRepertoire^112^. Clonal frequencies are based on cells remaining in the finalized CD8 T cell object.

Single-cell RNA-seq data from E22+ cells were processed as described above, including removing T cell receptor genes (117 genes). Dimensionality reduction and clustering with 30 PCs and a resolution of 1.0 was used to remove clusters of contaminating cells with high expression of *Ncr1* and *Cd74*. The remaining cells were clustered with 30 PCs and a resolution of 0.6, and a cluster of cells with a high fraction of mitochondrial reads was removed. The final CD8 T cell object was rescaled. The final object was mapped to the scTCR-seq UMAP using ProjecTILs version 3.0.1^39^, and cells were assigned to clusters based on the mapping. The *combineTCR* function from scRepertoire^112^ was used to combine TCR information with expression. Clonal assignments were made using the nucleotide sequences of cells with at least one alpha and one beta chain sequence detected. The mean hill diversity index was calculated using *alphaDiversity* from alakazam^113^ with a diversity order (q) of 1, a minimum of 100 cells per sample, and 100 bootstraps.

### Proteomic analysis

Tumor samples were collected on day 7 after the initiation of treatment. Tumors were snap-frozen in liquid nitrogen. Samples were homogenized in PBS and then shipped frozen to Biognosys (Biognosys AG, Wagistrasse 21, 8952 Schlieren, Switzerland) for analysis. Samples were prepared for mass spectrometry using Biognosys’ optimized protocol: lysate protein concentration was determined; Trypsin digestion was performed, followed by C18 purification of peptides and the quantification of final peptide concentration. A sample-specific spectral library was generated by creating two sample pools from aliquots of each sample (control plus anti-PD-L1, anti-TGFβ plus combo). High-pH reversed-phase chromatography (HPRP) fractionation of each pool (4 fractions) was performed, followed by shotgun LC-MS/MS of HPRP fractions (total 8 measurements) for spectral library generation and quality control. In all, 2 µg peptide digest was injected onto an Orbitrap Fusion Lumos mass spectrometer coupled to an Easy nLC 1200 (Thermo Fisher Scientific, San Jose, CA) for data-independent protein profiling. Peptides were separated over a 120 min chromatographic gradient followed by targeted data extraction. Data analysis was performed using Spectronaut Pulsar Software (Biognosys, Zurich, Switzerland). Differentially abundant proteins across conditions were visualized using TIBCO® Spotfire® 7.8.0 HF-002.

Proteins with an adjusted p value less than 0.05 were extracted and ranked from the highest to lowest fold change. The genes encoding these significantly changed proteins were identified, and markers genes of single-cell cluster T1 (*FindMarkers*, adj. *P* < 0.05) were used to calculate enrichment scores in the ranked proteomics dataset with fgsea (*fgseaSimple*).

### Reporting summary

Further information on research design is available in the Nature Portfolio Reporting Summary linked to this article.

### Supplementary information

Supplementary Information

Description of Additional Supplementary Files

Supplementary Data 1

Supplementary Data 2

Supplementary Data 3

Supplementary Data 4

Supplementary Data 5

Supplementary Data 6

Supplementary Data 7

Reporting Summary

### Source data

Source Data

## Data Availability

Bulk and scRNA-seq datasets data generated in this study have been deposited in the ArrayExpress database under accession codes E-MTAB-13072 (scRNA-seq), E-MTAB-13073 (scRNA/TCR-seq), and E-MTAB-13079 (bulk RNA-seq). Proteomics data in this study have been deposited in the MassIVE database under accession code MSV000092193. The IMvigor210 publicly available data used in this study are available in the European Genome-Phenome Archive under accession code EGAS00001002556. The TCGA publicly available Pan-Cancer mRNA data used in this study are available on UCSC Xena under dataset ID EB + +AdjustPANCAN_IlluminaHiSeq_RNASeqV2.geneExp.xena [https://xenabrowser.net/datapages/?cohort=TCGA%20Pan-Cancer%20(PANCAN)]. The remaining data are available within the Article, Supplementary Information, or Source Data file. Source data are provided with this paper.

## Code availability

No new algorithms were developed for this manuscript. Analysis code is available through the Open Science Framework (OSF) project with ID vmb96.
