# Supplementary material for: Combined PD-L1/TGFβ blockade allows expansion and differentiation of stem cell-like CD8 T cells in immune excluded tumors
Source: Nat Commun. 2023 Aug 5;14:4703. doi: 10.1038/s41467-023-40398-4 (PMC10404279; doi:10.1038/s41467-023-40398-4)
Supplement: Supplementary file 1 — Supplementary Information [file 41467_2023_40398_MOESM1_ESM.pdf]

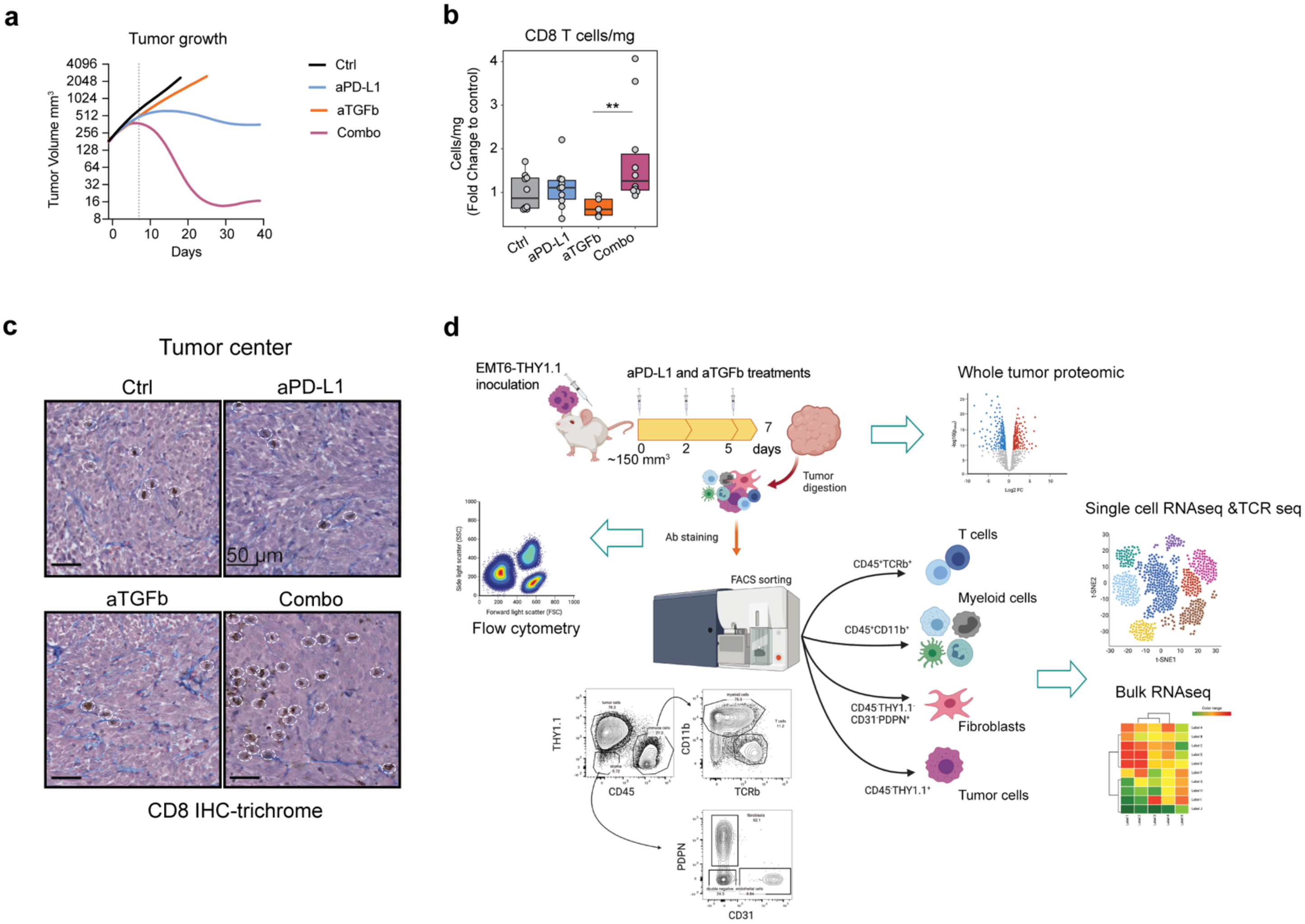

Supplementary Figure 1. EMT6 tumor growth curves and experimental layout.

(**a**) Group fit curves of EMT6 tumor volume when treated with anti-PD-L1 and anti-TGFβ alone or in combination from figure 1a. Representative experiment (10 animals, see Supplementary figure 1 for individual animal curves). Dashed line indicates day 7 after initiation of treatment.

(**b**) Tumor CD8 T cell quantification by flow cytometry (n=10 per group of treatment; data from two independent experiments; data are expressed as FC compared to the median of the control group across groups (x-axis)). Adjusted P value ** = 0.00855 Dunn's test (two-sided) with Benjamini-Hochberg multiple testing correction unless differently specified. Whiskers represent the minimum and maximum (unless points extend 1.5 * IQR from the hinge, then shown as individual points), the box represents the interquartile range, and the center line represents the median.

(**c**) Representative CD8 IHC staining of tumor center that is quantified in figure 1d. White dashed circles highlight CD8 T cells.

(**d**) Experimental layout. EMT6-THY1.1 tumor cells were inoculated in the mammary fat pad, treatments started when tumor size reached ~150mm3. At 7 days after initiation of treatment tumors were collected and analyzed by whole tumor proteomic, flow cytometry, or sc-RNA/TCRseq and bulk RNAseq on sorted populations (T cells, Myeloid cells, Fibroblasts and Tumor cells). Created with BioRender.com.

Source data are provided as a Source Data file. Ctrl = control (anti-GP120), aPD-L1 = anti-PD-L1, aTGFb = anti-TGFβ, combo = combination anti-PD-L1+ anti-TGFβ.

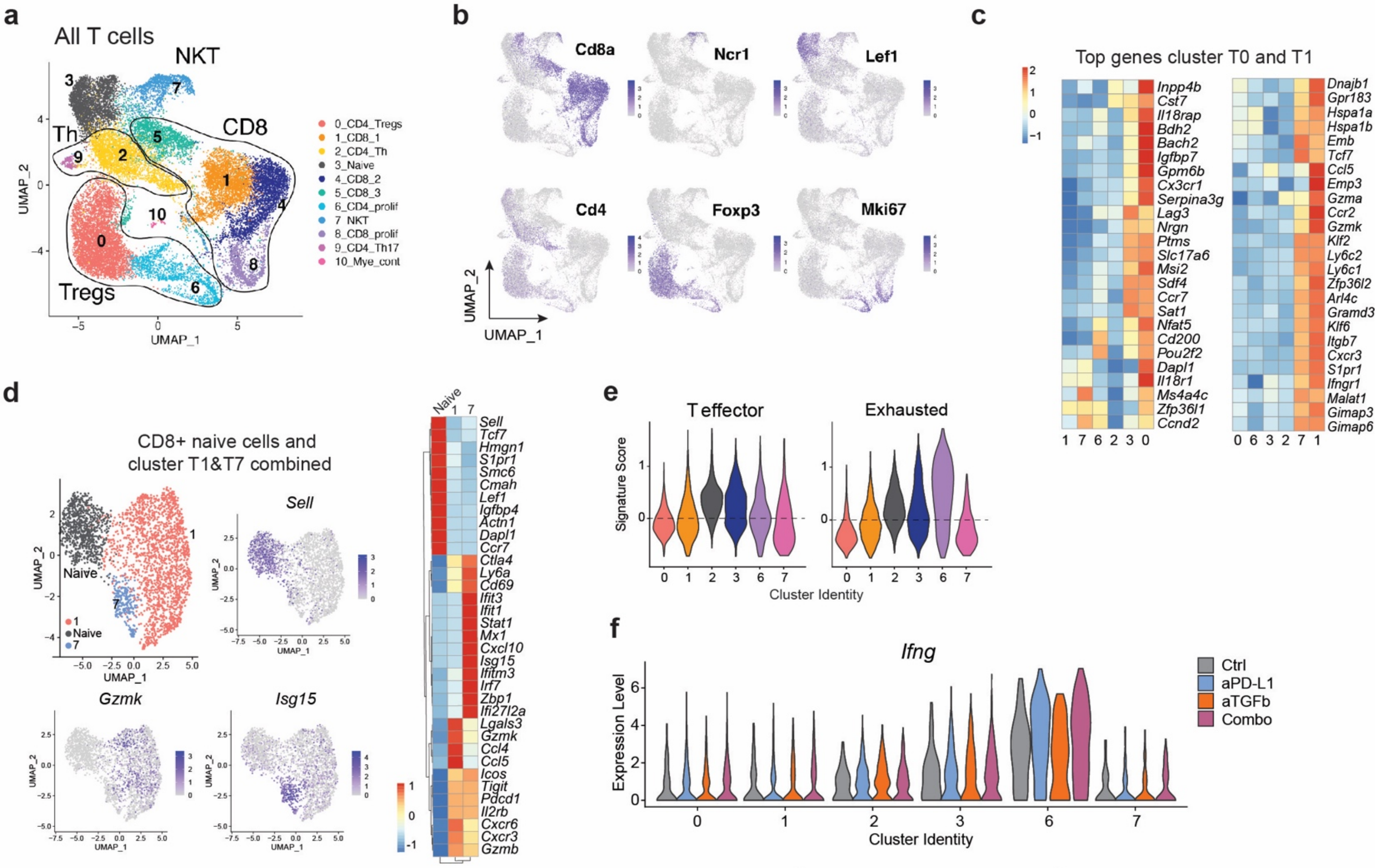

g

Pathway	Genes enriched in cluster T0	Reference
TGFb signaling	*Timp2, Igfbp7, Gpm6b, Nt5e lack of Gzmb expression*	Gomes et al., 2012[1], Rao et al., 2014[2]; Zhang et al., 2018[3]; Kordaß et al.2018[4]; Chen et al. 2019[5]; Thomas and Massagué, 2005[6]
Apoptosis	*Igfbp7, Anxa5, Dapl1 and Bdh2*	Verhagen et al., 2014[7]; Wajapeyee et al., 2008[8]; Koopman et al., 1994[9]; Liu et al., 2020[10]
Deletional tolerance	*Gpm6b, Tox, Nrn1*	Parish et al., 2009[11]
Anergy	*Gpm6b, Lag3, CD200, Alcam, Timp2*	Martinez et al., 2015[12]
Contraction phase	*Il18rap, Il18r1 Bach2, Serpina3g, Cx3cr1, Ccr7*	Haring and Harty, 2009[13]; Roychoudhuri et al., 2016[14]; Byrne et al., 2012[15]; Li et al., 2014[16]; Gerlach et al., 2016[17]; Förster et al., 2008[18]

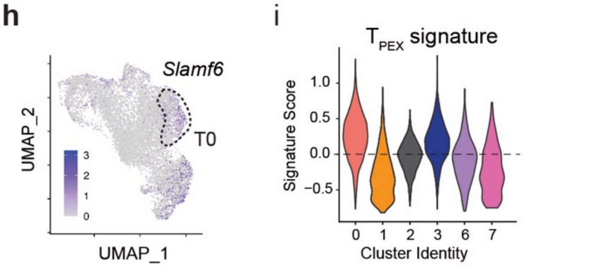

Supplementary Figure 2. CD8 T cell scRNA-seq filtering and downstream analysis.

(**a**) UMAP of 25,063 T cells (dots) colored by cluster.

(**b**) Expression of selected genes visualized on UMAP for identification of main T cells types.

(**c**) Relative average expression of the top 25 genes expressed by CD8 T cell clusters T0 and T1 from figure 2a.

(**d**) (Left) UMAP of CD8 naive T cell together with cells from clusters T1 and T7 from figure 2a. (Right) relative average gene expression of selected genes.

(**e**) T effector and exhausted signature enrichment scores in clusters from figure 2a.

(**f**) Expression level of *Ifng* per cluster from figure 2a seperated by treatment.

(**g**) Classification of genes enriched in cluster T0 with references.

(**h**) UMAP as in figure 2a, here colored by *Slamf6* expression.

(**i**) T_{PEX} gene signature enrichment in CD8 T cells clusters.

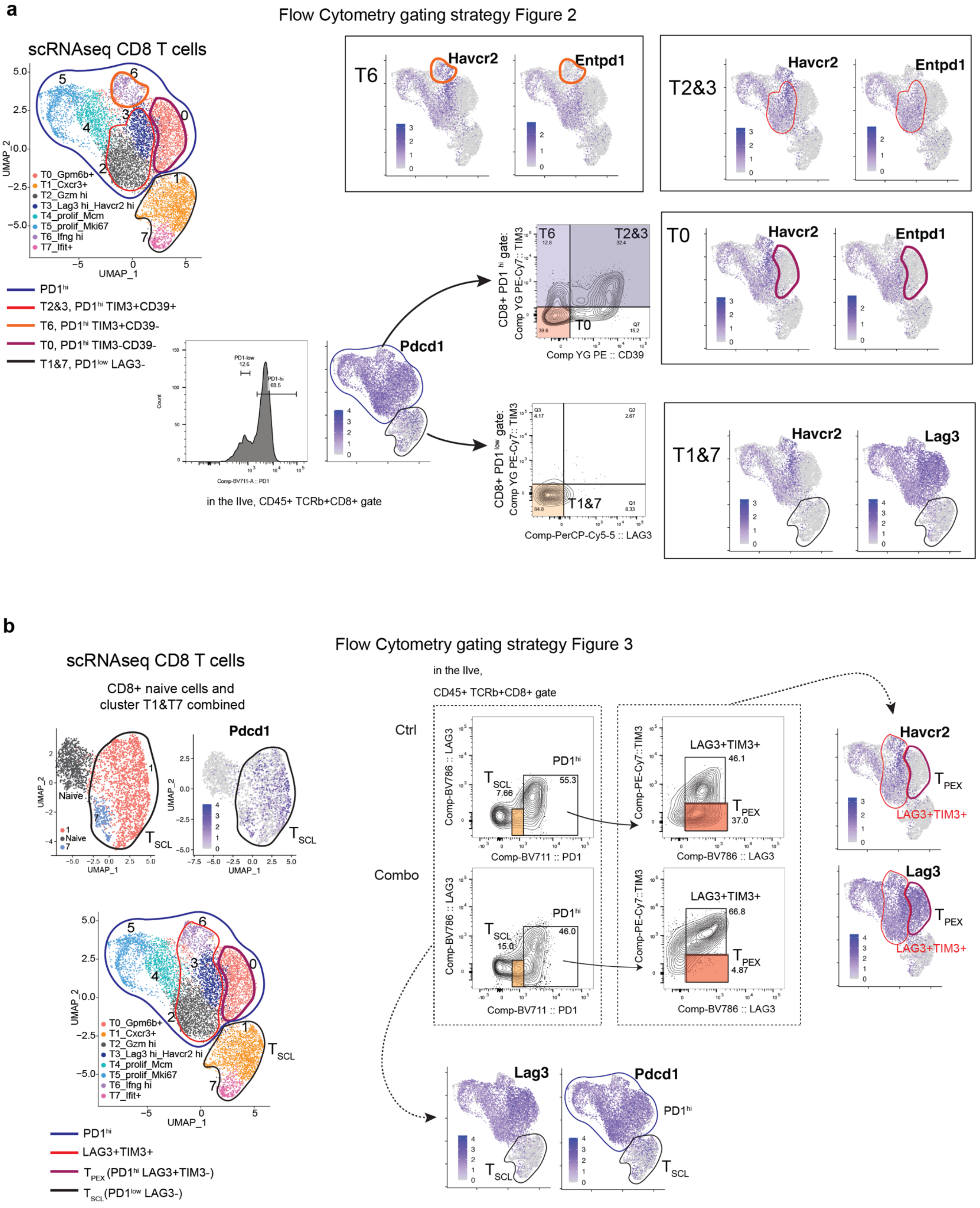
a
Flow Cytometry gating strategy Figure 2
scRNAseq CD8 T cells
T0_Gpm6b+
T1_Cxcr3+
T2_Gzm hi
T3_Lag3 hi_Havcr2 hi
T4_prolif_Mcm
T5_prolif_Mki67
T6_Ifng hi
T7_Ifit+
UMAP_1
UMAP_2
PD1hi
T2&3, PD1hi TIM3+CD39+
T6, PD1hi TIM3+CD39-
T0, PD1hi TIM3-CD39-
T1&7, PD1low LAG3-
T6
Havcr2
Entpd1
T2&3
T0
T1&7
Lag3
Pdcd1
PD1-low
12.6
PD1-hi
69.5
Count
Comp-BV711-A :: PD1
in the live, CD45+ TCRb+CD8+ gate
CD8+ PD1 hi gate: Comp YG PE-Cy7:: TIM3
Comp YG PE :: CD39
12.8
32.4
39.6
Q7
15.2
CD8+ PD1 low gate: Comp YG PE-Cy7:: TIM3
Comp-PerCP-Cy5-5 :: LAG3
Q3
4.17
Q2
2.67
84.8
Q1
8.33
b
Flow Cytometry gating strategy Figure 3
scRNAseq CD8 T cells
CD8+ naive cells and
cluster T1&T7 combined
Naive
TSCL
in the live,
CD45+ TCRb+CD8+ gate
Ctrl
Combo
Comp-BV786 :: LAG3
Comp-BV711 :: PD1
PD1hi
55.3
7.66
46.0
15.0
Comp-PE-Cy7::TIM3
LAG3+TIM3+
46.1
66.8
TPEX
37.0
4.87
LAG3+TIM3+
TPEX (PD1hi LAG3+TIM3-)
TSCL (PD1low LAG3-)

Supplementary Figure 3. Development of flow cytometry gating strategy.

(**a**) Gating strategy used to validate scRNAseq clusters by flow cytometry in figure 2. Within the live, CD45+TCRb+CD8+ gate two populations are distinguished: PD1hi and PD1low. Within the PD1hi population 3 different CD8 T cell populations are identified: T0, TIM3-CD39-; T6, TIM3+CD39-; and T2&3, TIM3+CD39+. The PD1low population is also LAG3- (T1&7).

(**b**) The top left panel shows the difference in PD1 expression between T_{SCL} and naive CD8 T cells by scRNAseq (naive cells are PD1 negative). Here, the gating strategy used to quantify T_{PEX} and T_{SCL} by flow cytometry in figure 3 and Supplementary figure 4, as well example plots from control and combo treated groups are shown. Within the live, CD45+TCRb+CD8+ gate two populations are gated: PD1hi and PD1lowLAG3- (T_{SCL}). Within the PD1hi population two different CD8 T cells populations are identified: T_{PEX} (LAG3+TIM3-) and TIM3+LAG3+ cells. The percentages of T_{PEX} and T_{SCL} in Supplementary figure 4 were calculated within the CD8+ T cells excluding naive (PD1 negative) cells.

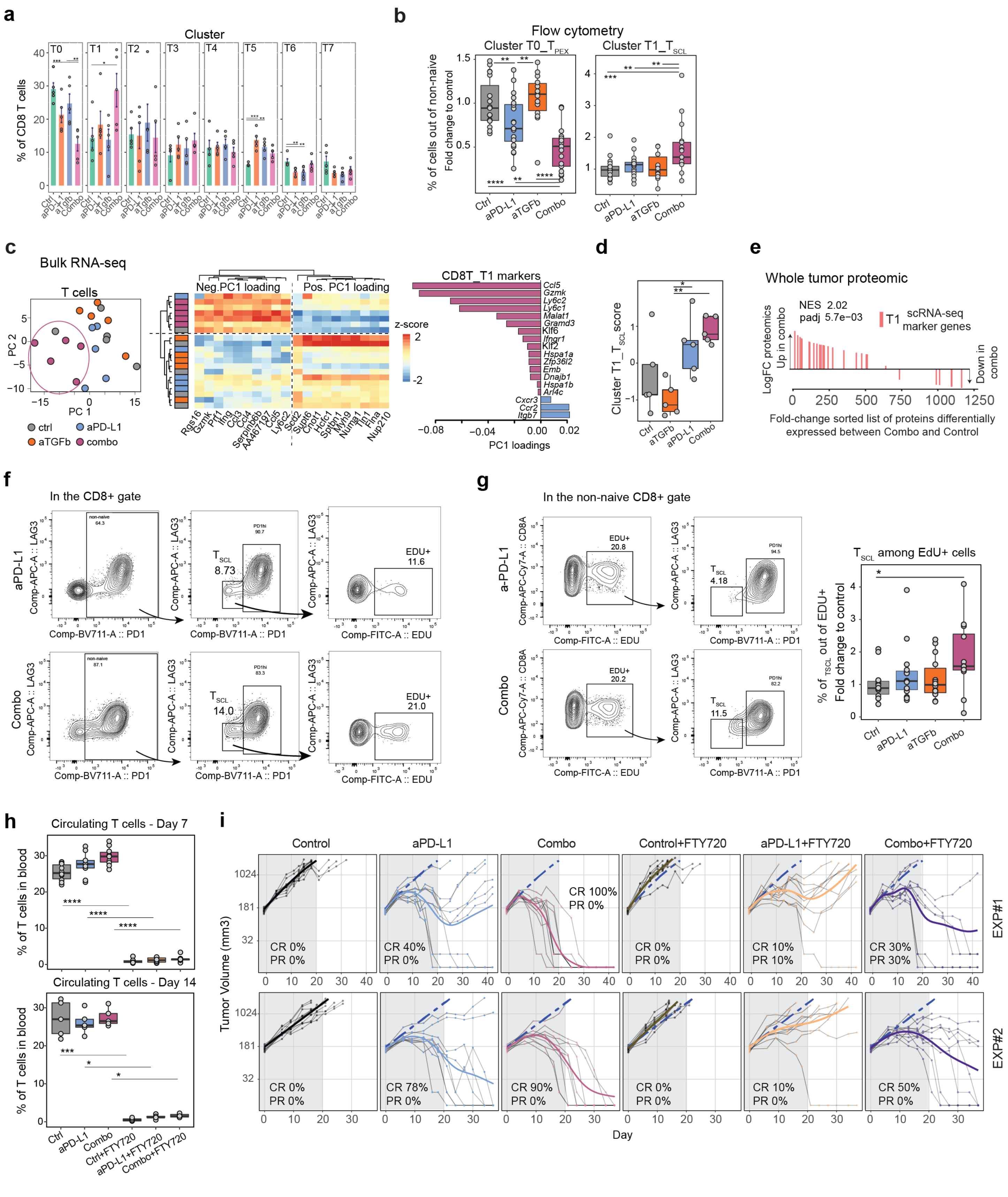
a
Cluster
T0 T1 T2 T3 T4 T5 T6 T7
% of CD8 T cells
Ctrl aPD-L1 aTgfb Combo
b
Flow cytometry
Cluster T0_TPEX
Cluster T1_TSCL
% of cells out of non-naive
Fold change to control
Ctrl aPD-L1 aTGFb Combo
c
Bulk RNA-seq
T cells
PC 2
PC 1
ctrl aPD-L1 aTGFb combo
Neg.PC1 loading
Pos. PC1 loading
z-score
CD8T_T1 markers
Ccl5 Gzmk Ly6c2 Ly6c1 Malat1 Gramd3 Klf6 Ifngr1 Klf2 Hspa1a Zfp36l2 Emb Dnajb1 Hspa1b Arl4c Cxcr3 Ccr2 Itgb7
PC1 loadings
d
Cluster T1_TSCL score
Ctrl aTGFb aPD-L1 Combo
e
Whole tumor proteomic
NES 2.02
padj 5.7e−03
T1 scRNA-seq marker genes
LogFC proteomics Up in combo
Down in combo
Fold-change sorted list of proteins differentially expressed between Combo and Control
f
In the CD8+ gate
aPD-L1
Combo
non-naive
PD1hi
TSCL
EDU+
Comp-APC-A :: LAG3
Comp-BV711-A :: PD1
Comp-FITC-A :: EDU
g
In the non-naive CD8+ gate
a-PD-L1
Combo
Comp-APC-Cy7-A :: CD8A
Comp-FITC-A :: EDU
Comp-APC-A :: LAG3
Comp-BV711-A :: PD1
TSCL among EdU+ cells
% of TSCL out of EDU+
Fold change to control
Ctrl aPD-L1 aTGFb Combo
h
Circulating T cells - Day 7
Circulating T cells - Day 14
% of T cells in blood
Ctrl aPD-L1 Combo Ctrl+FTY720 aPD-L1+FTY720 Combo+FTY720
i
Control aPD-L1 Combo Control+FTY720 aPD-L1+FTY720 Combo+FTY720
Tumor Volume (mm3)
Day
EXP#1
EXP#2
CR 0% PR 0%
CR 40% PR 0%
CR 100% PR 0%
CR 0% PR 0%
CR 10% PR 10%
CR 30% PR 30%
CR 0% PR 0%
CR 78% PR 0%
CR 90% PR 0%
CR 0% PR 0%
CR 10% PR 0%
CR 50% PR 0%

Supplementary Figure 4. Changes in CD8 T cell phenotypes induced by anti-PD-L1 and anti-TGFβ treatment.

(**a**) Mean frequency of CD8 T cell clusters from figure 2a by treatment group (n=5 per group). Error bars represent SEM.
(**b**) Flow cytometry quantification of CD8 T_{SCL} (PD1lowLAG3-) and TPEX cells (PD1hiLAG3+TIM3-) (percentage of cell in the non-naive CD8 T cells population, fold-change over average of the control group: y-axis; groups: x-axis; data from three independent experiments, ctrl n=19, aPD-L1 n=20, aTGFb n=14, combo n=17).
(**c**) Bulk RNA-seq analysis on sorted T cells (n=5 per group). (Left) PCA analysis of T cells by treatment. (Middle) Heatmap of top 10 genes positively and negatively loading PC1. (Right) PC1 loadings of indicated T_{SCL} marker genes.
(**d**) Cluster T1 signature score in bulk RNAseq performed on sorted T cells by treatment (n=5 per group).
(**e**) Proteomic analysis on whole tumor (n=10 per group). Gene set enrichment analysis on proteins rank ordered by fold-change between combination and control highlighting proteins encoding T_{SCL} marker genes.
(**f**) Gating strategy used for the flow cytometric analysis of EdU+ T_{SCL} presented in figure 3g. Representative plots from anti-PD-L1 and combo treated groups.
(**g**) (Left) Gating strategy used for the analysis presented in the right panel. (Right) Flow cytometry quantification of T_{SCL} in the EdU+ CD8 T cells (percentage of non-naive CD8 T cells, fold-change over average of the control group: y-axis; groups: x-axis; data from two independent experiments, ctrl n=14, aPD-L1 n=15, aTGFb n=14, combo n=12).
(**h**) Percentage of T cells circulating in blood at days 7 and 14 after initiation of FTY720 treatment (% of total CD45+: y-axis; groups: x-axis; two independent experiments, n = 10 for all groups for day 7, n = 5 for all groups for day 14). Here only significant comparisons between a treatment and their respective counterpart with FTY720 are shown.
(**i)** EMT6 tumor volume showing the individual animal curves relative to figure 3h. Grey shades indicate treatment duration. Two independent experiments are shown.

Source data are provided as a Source Data file. Ctrl = control (anti-GP120), aPD-L1 = anti-PD-L1, aTGFb = anti-TGFβ, combo = combination anti-PD-L1+ anti-TGFβ; Adj P values *<0.1, **<0.05, ***<0.01, ****<0.001 Dunn's test (two-sided) with Benjamini-Hochberg multiple testing correction unless differently specified. Adjusted P values for **a**: T0: 0.005517513 (ctrl vs combo**),** 0.030889647 (aTGFb vs combo); T1: 0.08364055 (ctrl vs combo); T5: 0.003744252 (aPD-L1 vs ctrl), 0.016331945 (aTGFb vs ctrl); T6: 0.03266389 (aPD-L1 vs ctrl), 0.02644396 **(**aTGFb vs ctrl). **b**: T_{PEX}: 0.0000275 (ctrl vs combo), 0.0212 (aPD-L1 vs combo), 0.000014 (aTGFb vs combo), 0.0433 (ctrl vs aPD-L1), 0.0205 (aTGFb vs aPD-L1); T_{SCL}: 0.00291 (ctrl vs combo), 0.0374 (aPD-L1 vs combo), 0.0207 (aTGFb vs combo). Adjusted P values for **d**: 0.02337900 (aTGFb vs combo), 0.08523774 (aTGFb vs aPD-L1). Adjusted P values for **g**: 0.0751 (ctrl vs combo). Adjusted P values for **h,** day 7: 0.000634 (ctrl vs ctrl+FTY720), 0.000623 (aPD-L1 vs aPD-L1+FTY720), 0.000193 (combo vs combo+FTY720); day 14: 0.00245 (ctrl vs ctrl+FTY720), 0.0529 (aPD-L1 vs aPD-L1+FTY720), 0.0617 (combo vs combo+FTY720). For **b, d, g, h** whiskers represent the minimum and maximum (unless points extend 1.5 * IQR from the hinge, then shown as individual points), the box represents the interquartile range, and the center line represents the median.

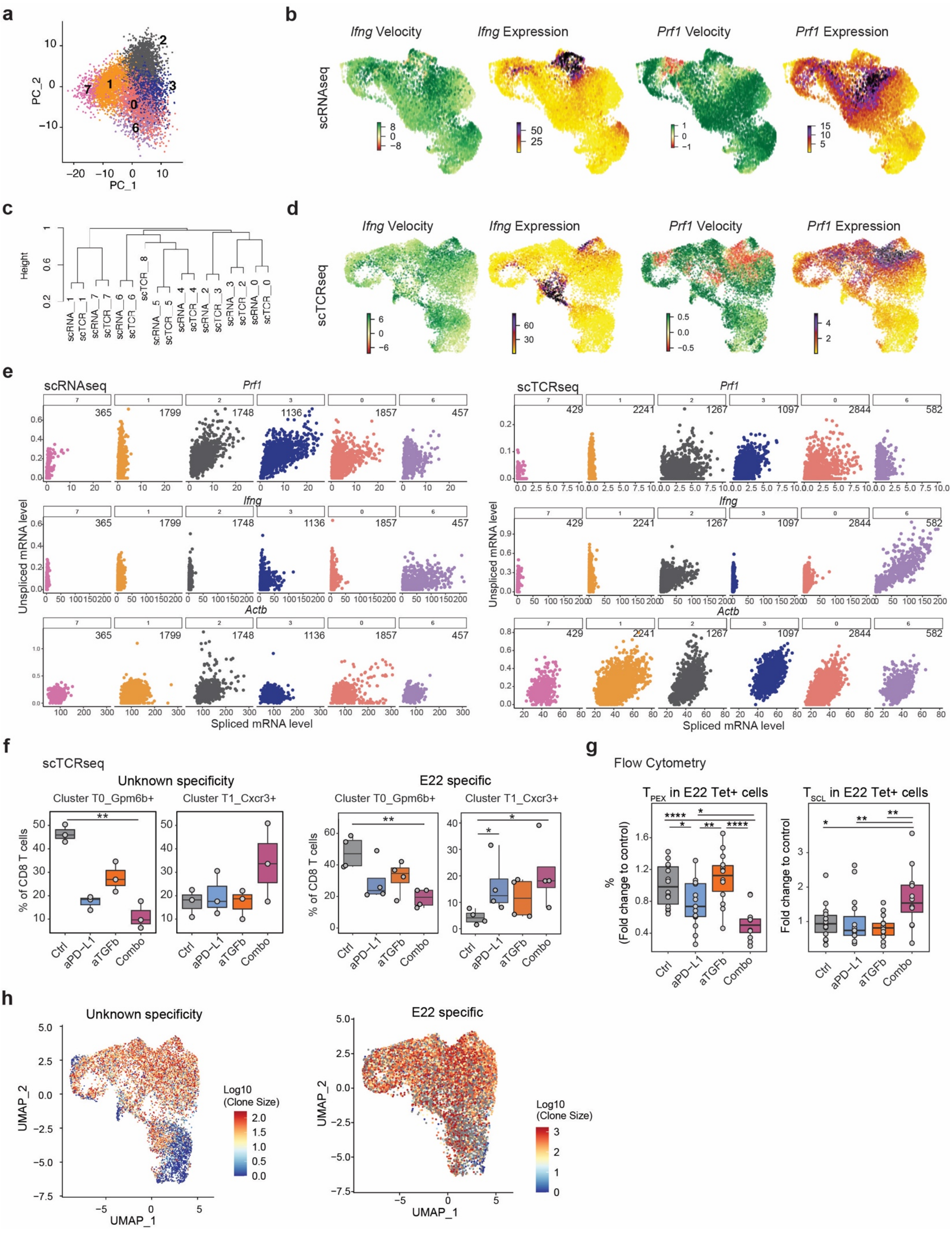

a
PC_2
PC_1
b
Ifng Velocity
Ifng Expression
Prf1 Velocity
Prf1 Expression
scRNAseq
c
Height
scRNA_1
scTCR_1
scRNA_7
scTCR_7
scRNA_6
scTCR_6
scTCR_8
scRNA_5
scTCR_5
scRNA_4
scTCR_4
scRNA_2
scTCR_3
scRNA_3
scTCR_2
scRNA_0
scTCR_0
d
scTCRseq
e
scRNAseq
scTCRseq
Prf1
Ifng
Actb
Unspliced mRNA level
Spliced mRNA level
f
scTCRseq
Unknown specificity
E22 specific
Cluster T0_Gpm6b+
Cluster T1_Cxcr3+
% of CD8 T cells
Ctrl
aPD−L1
aTGFb
Combo
g
Flow Cytometry
T PEX in E22 Tet+ cells
T SCL in E22 Tet+ cells
%
(Fold change to control)
Fold change to control
h
Unknown specificity
E22 specific
Log10
(Clone Size)
UMAP_1
UMAP_2

Supplementary Figure 5. scRNA/TCR-seq analysis.

(**a**) Cells from figure 2a, here in PCA space.

(**b**) Visualization of *Ifng* and *Prf1* velocity (left) and expression (right) on the UMAP for scRNA-seq dataset as in figure 2a.

(**c**) Dendrogram of CD8 T cell clusters in two single-cell datasets: scRNA and unsorted scTCR-seq from figures 2a and 4d (left), respectively.

(**d**) Visualization of *Ifng* and *Prf1* velocity (left) and expression (right) on the UMAP for the unsorted scTCR-seq dataset as in figure 4d (left).

(**e**) Quantification of spliced vs unspliced *Ifng*, *Prf1*, and *Actb* (as a reference gene) transcripts using first order moments. Left is for the scRNA-seq dataset, right is for the unsorted scTCR-seq dataset. Number of cells in each plot are in the upper right corner.

(**f**) ScTCR-seq cluster T0 and T1 frequency quantification in unknown specificity (left; n=3 animals per treatment group) and E22 specific cells (right; n=4 animals per treatment group).

(**g**) Flow cytometry quantification of CD8 T_{SCL} (PD1lowLAG3-) and T_{PEX} cells (PD1hiLAG3+TIM3-) within the E22 specific CD8 T cells (percentage of cells, fold-change over average of the control group: y-axis; groups: x-axis; data from two independent experiments, Ctrl n=14; aPD-L1 n=15; aTGFb n=14; Combo n=12).

(**h**) UMAP of CD8 T cells colored by clone size for unknown specificity (left) and E22 specific cells (right).

Source data are provided as a Source Data file. Ctrl = control (anti-GP120), aPD-L1 = anti-PD-L1, aTGFb = anti-TGFβ, combo = combination anti-PD-L1+ anti-TGFβ; Adj P values *<0.1, **<0.05, ***<0.01, ****<0.001 Dunn's test (two-sided) with Benjamini-Hochberg multiple testing correction unless differently specified. Adjusted P values for **f**: Unsorted Cluster T0_Gpm6b+ 0.0194 (Ctrl vs Combo). E22 Cluster T0_Gpm6b+ 0.0286 (Ctrl vs Combo); E22 Cluster T1_Cxcr3+ 0.0560 (Ctrl vs Combo), 0.0938 (Ctrl vs aPD-L1). Adjusted P values for **g**: T_{PEX} 0.000527 (ctrl vs combo), 0.0772 (aPD-L1 vs combo), 0.000092 (aTGFb vs combo), 0.0626 (ctrl vs aPD-L1), 0.0216 (aTGFb vs aPD-L1); T_{SCL} 0.0568 (ctrl vs combo), 0.0209 (aPD-L1 vs combo), 0.0254 (aTGFb vs combo). For **f and g** whiskers represent the minimum and maximum (unless points extend 1.5 * IQR from the hinge, then shown as individual points), the box represents the interquartile range, and the center line represents the median.

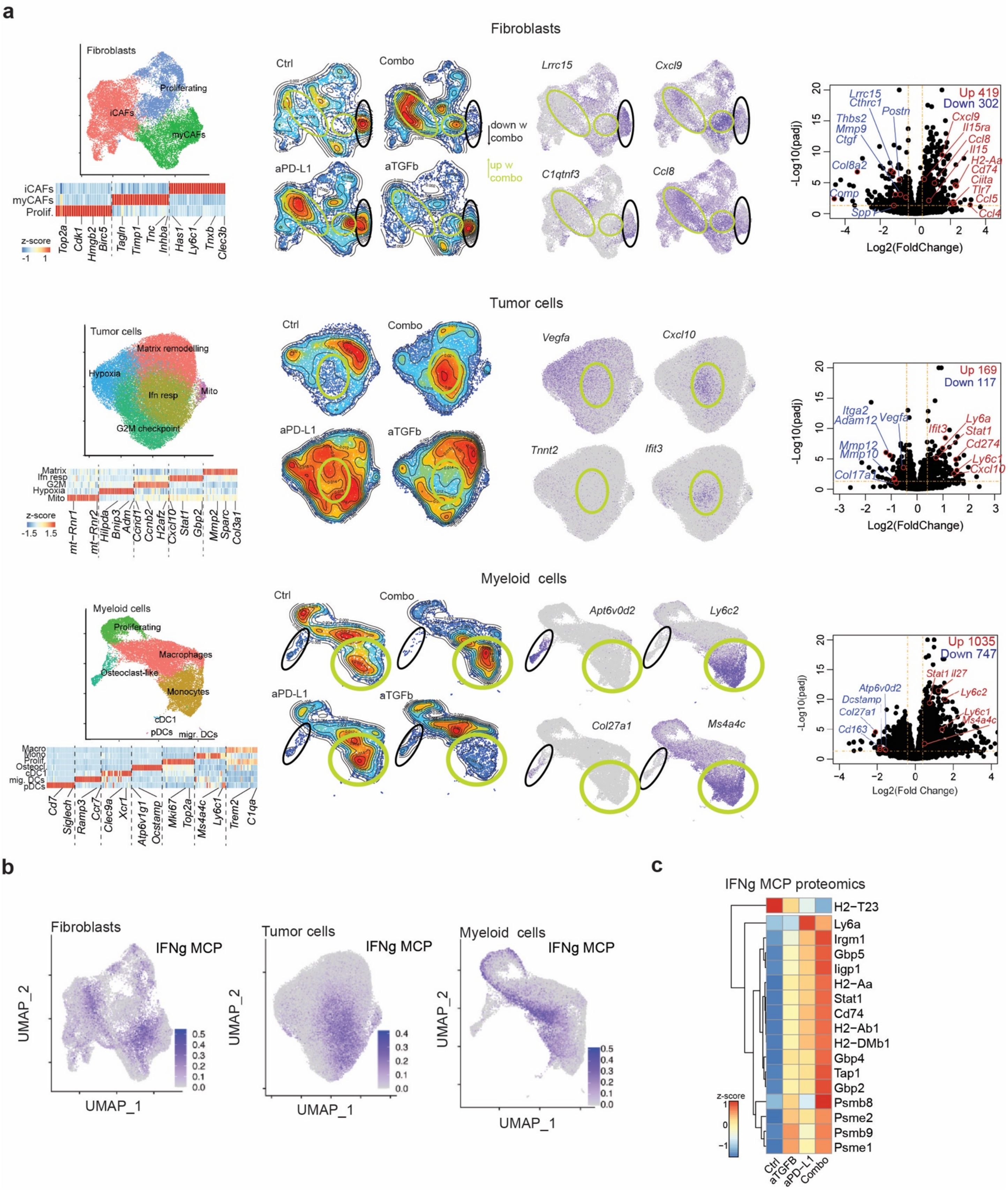
a
Fibroblasts
iCAFs
Proliferating
myCAFs
Prolif.
z-score
-1
1
Top2a
Cdk1
Hmgb2
Birc5
Tagln
Timp1
Tnc
Inhba
Has1
Ly6c1
Tnxb
Clec3b
Ctrl
Combo
aPD-L1
aTGFb
down w combo
up w combo
Lrrc15
Cxcl9
C1qtnf3
Ccl8
Up 419
Down 302
Lrrc15
Cthrc1
Postn
Thbs2
Mmp9
Ctgf
Col8a2
Comp
Spp1
Cxcl9
Il15ra
Ccl8
Il15
H2-Aa
Cd74
Ciita
Tlr7
Ccl5
Ccl4
-Log10(padj)
Log2(FoldChange)
Tumor cells
Matrix remodelling
Hypoxia
Ifn resp
Mito
G2M checkpoint
Matrix
Ifn resp
G2M
Hypoxia
Mito
z-score
-1.5
1.5
mt−Rnr1
mt−Rnr2
Hilpda
Bnip3
Adm
Ccnd1
Ccnb2
H2afz
Cxcl10
Stat1
Gbp2
Mmp2
Sparc
Col3a1
Vegfa
Cxcl10
Tnnt2
Ifit3
Up 169
Down 117
Itga2
Adam12
Vegfa
Mmp12
Mmp10
Col17a1
Ifit3
Ly6a
Stat1
Cd274
Ly6c1
Cxcl10
Myeloid cells
Proliferating
Macrophages
Osteoclast-like
Monocytes
cDC1
pDCs
migr. DCs
Macro
Mono
Prolif.
Osteocl.
cDC1
mig. DCs
pDCs
Cd7
Siglech
Ramp3
Ccr7
Clec9a
Xcr1
Atp6v1g1
Ocstamp
Mki67
Top2a
Ms4a4c
Ly6c1
Trem2
C1qa
Apt6v0d2
Ly6c2
Col27a1
Ms4a4c
Up 1035
Down 747
Atp6v0d2
Dcstamp
Col27a1
Cd163
Stat1 il27
Ly6c2
Ly6c1
Ms4a4c
Log2(Fold Change)
b
Fibroblasts
Tumor cells
Myeloid cells
IFNg MCP
UMAP_1
UMAP_2
c
IFNg MCP proteomics
H2−T23
Ly6a
Irgm1
Gbp5
Iigp1
H2−Aa
Stat1
Cd74
H2−Ab1
H2−DMb1
Gbp4
Tap1
Gbp2
Psmb8
Psme2
Psmb9
Psme1
z-score
Ctrl
aTGFB
aPD−L1
Combo

Supplementary Figure 6. scRNA-seq analysis of myeloid, tumor cells, and CAFs and proteomic validation.

(**a**) (1st column) UMAP of fibroblasts, tumor cells and myeloid cells (dots) colored by cluster. Markers for individual clusters are given by heatmaps on the bottom. (2nd column) The density of cells from each condition is indicated by color in the middle (blue: sparse; red: dense). (3rd column) Expression of indicated marker genes on the UMAP from the left. (4th column) Pseudo-bulk differential expression analysis comparing cells from the control to the anti-PD-L1 anti-TGFβ combination (n=5 per group). Yellow lines indicate significance thresholds: fold change, ± 0.4 and adjusted p-value< 0.05 (DEseq2), DEG: differentially expressed genes.

(**b**) UMAP as in (a) of fibroblast (left), tumor (middle), and myeloid cells (right), colored by IFNg MCP program activity.

(**c**) Proteomic analysis on whole tumor samples across conditions (n=10 per group). Heatmap represents z-scores of the relative abundance compared to control for proteins encoded by genes in the IFNg MCP program.

Ctrl = control (anti-GP120), aPD-L1 = anti-PD-L1, aTGFb = anti-TGFβ, combo = combination anti-PD-L1+ anti-TGFβ.

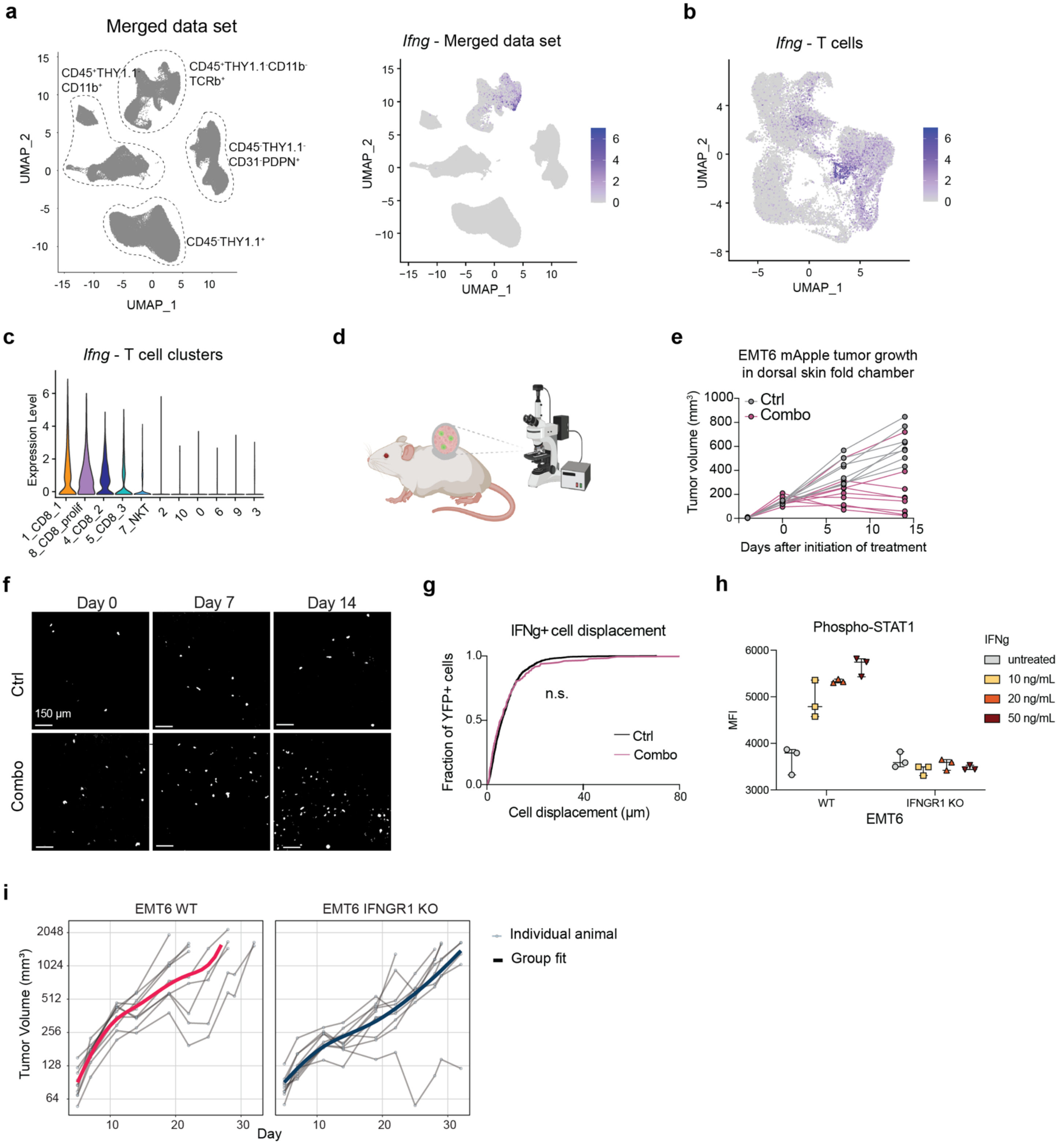

Supplementary Figure 7. Identification of CD8 T cells as the main IFNγ source in the TME and *in vivo* imaging.

(a) (Left) UMAP of merged data set with cells coming from all the sorted populations from the tumor. (Right) *Ifng* expression on merged data set.

(b) *Ifng* expression in T cell UMAP as in Supplementary figure 2a.

(c) Violin plot of *Ifng* expression in T cell clusters from Supplementary figure 2a.

(d) Dorsal skin fold chamber (DSFC) *in vivo* imaging set up. EMT6 mAPPLE tumor cells were implanted in the DSFC mounted on the back of YFP IFNγ reporter mice. Video recordings were made using 2-photon microscopy (created with biorender.com).
(e) Tumor growth of EMT6 mAPPLE in DSFC (data from one experiment, n=8 control group, n=9 combination group).
(f) Representative images of the IFNγ -YFP channel of one control and one combo treated tumors.
(g) IFNγ-YFP+ cell displacement at day 7-9 after initiation of treatment (Control: n = 179 cells from 8 mice; Combination: n = 2,310 cells from 7 mice (non-responders excluded); P value <0.0001, two-tailed Kolmogorov-Smirnov test).
(h) *In vitro* validation of the IFNGR1 KO EMT6 cells. WT and KO cells were treated for 30 minutes with IFNγ at the indicated concentrations. Cells were then harvested and STAT1 phosphorylation was assessed by flow cytometry (MFI = mean fluorescence intensity; n=3 technical replicates; middle line is median, error bars represent min to max values).
(i) *In vivo* tumor growth analysis of IFNGR1 KO EMT6 cells compared to WT. Individual animal curves (grey lines) and group fit curves (thick solid lines) are provided, n=10.

Source data are provided as a Source Data file. Ctrl = control (anti-GP120), combo = combination anti-PD-L1+ anti-TGFβ.

Supplementary Table 1: ScRNA-seq cell numbers.

Cell type	all	ctrl	aPD-L1	aTGFb	combo
T cells	25063	2463	6805	7687	8108
CD11b+ cells	26867	5469	6967	7880	6551
Fibroblasts	37773	7026	11471	7959	11317
Tumor cells	45024	7128	14416	12415	11065
**Total cells in study**	**119185**	**19141**	**35883**	**32190**	**31971**
**Sub populations:**	**all**	**ctrl**	**aPD-L1**	**aTGFb**	**combo**
Myeloid cells, neutrophils excluded	21601	4467	5786	6494	4854
CD8 T cells	9525	690	3087	2765	2983
Tregs	7190	659	2148	2199	2184
NKT	1390	174	408	352	456
CD4 T cells	3352	357	943	971	1081
Fibroblasts, filtered	22231	4081	7695	4208	6247

Supplementary Table 2: Gene signatures.

Signature name	Genes	Source
T_{SCM}	Tcf7 Sell Il2rb Cxcr3 Ly6a Bcl2 Klf2	Derived from<br>Zhang, Y at al. (2005) [19]<br>Gattinoni, L. et al. (2009)[20]<br>Graef, P. et al. (2014)[21]<br>Crompton, J. G. et al. (2016)[22]<br>Gattinoni, L., et al (2017)[23]
T effector	Cd8a Gzma Gzmb Prf1 Cxcl9 Cxcl10 Tbx21	Mariathasan, S. et al. (2018)[24]
Exhausted	Gzmb Sytl2 Ccl3 Ccl4 Cd244 Chn2 Cish Cxcr6 Fasl Plekhf1	Texhausted_list_6 from:<br>Beltra, J.-C. et al (2020)[25]
Cytotoxic	Fasl Gzmf Gzmc Gzmb Gzmd Prf1 Ifng Tnf Ccl4 Ccl3 Ccl5	Halle, S., et al. (2017)[26]<br>Allen, F. et al (2017)[27]<br>Bhat, P. et al (2017)[28]<br>Lieberman, J. (2003)[29]
T_{PEX}	Pdcd1 Tcf7 Lag3 Gpm6b Slamf6	Utzschneider, D. T. et al. (2016)[30]<br>Hudson, W. H. et al. (2019)[31]<br>Im, S. J et al. (2020)[32]<br>Siddiqui, I. et al. (2019)[33]<br>Andreatta, M. et al. (2021)[34]

Supplementary Table 3: T cells cluster frequency statistical analysis.

Cluster	Comparison	Z	P.unadj	P.adj
0	Combo-Ctrl	-3.31404	0.00092	0.005518
0	Combo-aPD-L1	-1.71047	0.087179	0.174357
0	Ctrl-aPD-L1	1.603568	0.108809	0.163214
0	Combo-aTGFb	-2.56571	0.010297	0.03089
0	Ctrl-aTGFb	0.748332	0.45426	0.45426
0	aPD-L1-aTGFb	-0.85524	0.392421	0.470905
1	Combo-Ctrl	2.458803	0.01394	0.083641
1	Combo-aPD-L1	1.603567	0.108809	0.217619
1	Ctrl-aPD-L1	-0.85524	0.392421	0.588631
1	Combo-aTGFb	2.031185	0.042236	0.126709
1	Ctrl-aTGFb	-0.42762	0.668929	0.668929
1	aPD-L1-aTGFb	0.427618	0.668929	0.802715
2	Combo-Ctrl	-0.64143	0.521245	1
2	Combo-aPD-L1	-0.37417	0.708281	1
2	Ctrl-aPD-L1	0.267261	0.789268	1
2	Combo-aTGFb	-0.58797	0.556549	1
2	Ctrl-aTGFb	0.053452	0.957372	0.957372
2	aPD-L1-aTGFb	-0.21381	0.830696	0.996835
3	Combo-Ctrl	1.389759	0.164602	0.987613
3	Combo-aPD-L1	0.748332	0.45426	0.908521
3	Ctrl-aPD-L1	-0.64143	0.521245	0.781868
3	Combo-aTGFb	1.17595	0.239615	0.718845
3	Ctrl-aTGFb	-0.21381	0.830696	0.830696
3	aPD-L1-aTGFb	0.427618	0.668929	0.802715
4	Combo-Ctrl	-0.16042	0.872553	0.872553
4	Combo-aPD-L1	-0.64167	0.521089	1
4	Ctrl-aPD-L1	-0.48125	0.630338	0.945507
4	Combo-aTGFb	-1.01597	0.309641	1
4	Ctrl-aTGFb	-0.85556	0.392243	1
4	aPD-L1-aTGFb	-0.37431	0.708176	0.849812
5	Combo-Ctrl	1.817376	0.069159	0.138319
5	Combo-aPD-L1	-1.60357	0.108809	0.163214
5	Ctrl-aPD-L1	-3.42094	0.000624	0.003744
5	Combo-aTGFb	-0.96214	0.335979	0.403175
5	Ctrl-aTGFb	-2.77952	0.005444	0.016332
5	aPD-L1-aTGFb	0.641427	0.521245	0.521245
6	Combo-Ctrl	-0.90869	0.363515	0.436218
6	Combo-aPD-L1	1.870829	0.061369	0.122738
6	Ctrl-aPD-L1	2.779517	0.005444	0.032664
6	Combo-aTGFb	1.710472	0.087179	0.130768
6	Ctrl-aTGFb	2.61916	0.008815	0.026444
6	aPD-L1-aTGFb	-0.16036	0.8726	0.8726
7	Combo-Ctrl	-1.38976	0.164602	0.329205
7	Combo-aPD-L1	0.587975	0.556549	0.667859
7	Ctrl-aPD-L1	1.977733	0.047959	0.143876
7	Combo-aTGFb	0.694879	0.487131	0.730697
7	Ctrl-aTGFb	2.084638	0.037102	0.222613
7	aPD-L1-aTGFb	0.106905	0.914865	0.914865

Supplementary Table 4: Antibody list.

Antibody	Origin	Identifier
CD11b BUV395, clone M1/70	BD Bioscience	Cat # 563553
CD11b BV650, clone M1/70	Biolegend	Cat # 101259
CD11b BV785, clone M1/70	Biolegend	Cat # 101243
CD19 BV650, clone 6D5	Biolegend	Cat # 115541
CD31 BUV737, MEC13.3	BD Bioscience	Cat # 612802
CD39 PE, clone Duha59	Biolegend	Cat # 143803
CD44 FITC, clone IM7	Biolegend	Cat # 103006
CD45 BV605, clone 30-F11	BD Bioscience	Cat # 563053
CD45 BUV395, clone 30-F11	BD Bioscience	Cat # 564279
CD45 PE-Cy7, clone 30-F11	Biolegend	Cat # 103114
CD8 APC-Cy7, clone 53-6.7	Biolegend	Cat # 100714
CD4 BV711, clone RM4-5	Biolegend	Cat # 100557
CD90.2 BV785, clone 30-H12	Biolegend	Cat # 105331
F4-80 BV711, clone BM8	Biolegend	Cat # 123147
Granzyme B FITC, clone NGZB	Thermo Fisher Scientific	Cat # 11-8898-82
IFNg PE-Cy7, clone XMG1.2	Biolegend	Cat # 505825
LAG3 BV785, clone C9B7W	Biolegend	Cat # 125219
LAG3 Percp-Cy5.5, clone C9B7W	Biolegend	Cat # 125211
LAG3 APC, clone C9B7W	Thermo Fisher Scientific	Cat # 17-2231-82
LY6C Percp-Cy5.5, clone HK1.4	Biolegend	Cat # 128012
LY6G APC-Cy7, clone 1A8	Biolegend	Cat # 127624
NK1.1 BV650, clone PK136	Biolegend	Cat # 108736
PD-1 BV711, clone 29F.1A12	Biolegend	Cat # 135231
PDPN APC-Cy7, clone 8.1.1	Biolegend	Cat # 127418
PDPN PE, clone 8.1.1	Biolegend	Cat #127408
TCF1 AF647, clone C63D9	Cell Signaling	Cat # 6709S
TCRb APC, clone H57-597	Thermo Fisher Scientific	Cat # 17-5961-82
TCRb PE, clone H57-597	Biolegend	Cat # 109208
THY1.1 AF488, clone OX-7	Biolegend	Cat # 202506
TIM3 PE-Cy7, clone RMT3-23	Biolegend	Cat # 119716
PE E22 tetramer (H-2Kd, peptide seq: RYAQAFTLL)	MBL International	Cat # TBCM3-KDI-1
PE LCMV tetramer (H-2Kd)	MBL International	Cat # TS-M514-1
P-STAT1 (Tyr701) AF488, rabbit mAb clone 58D6	Cell Signaling	Cat # 9174S
TotalSeq™-B0301	Biolegend	Cat #155831
TotalSeq™-B0302	Biolegend	Cat #155833
TotalSeq™-B0303	Biolegend	Cat #155835
TotalSeq™-B0304	Biolegend	Cat #155837
TotalSeq™-B0305	Biolegend	Cat #155839
TotalSeq™-C0301	Biolegend	Cat #155861
TotalSeq™-C0302	Biolegend	Cat #155863
TotalSeq™-C0303	Biolegend	Cat #155865
TotalSeq™-C0304	Biolegend	Cat #155867
TotalSeq™-C0305	Biolegend	Cat #155869

TotalSeq™-C0306	Biolegend	Cat #155871
Fc block, clone 2.4G2	BD Bioscience	Cat #553142
biotinylated goat anti-hamster IgG	Vectorlabs	BA-9100
hamster anti-mouse CD8a, clone 1.21E3.1.3	Genentech	
anti-mouse PD-L1, clone 6E11	Genentech	
Anti-TGFb, clones 1D11 and 1D11.V8.13.EG	Genentech	
Anti-gp120	Genentech	
Anti-mouse IFNg, clone Clone XMG1.2	BioXcell	Cat # BP0055

Supplementary references

1. Gomes, L. R., Terra, L. F., Wailemann, R. A., Labriola, L. & Sogayar, M. C. TGF-β1 modulates the homeostasis between MMPs and MMP inhibitors through p38 MAPK and ERK1/2 in highly invasive breast cancer cells. *Bmc Cancer* 12, 26 (2012).

2. Rao, C. *et al.* High Expression of IGFBP7 in Fibroblasts Induced by Colorectal Cancer Cells Is Co-Regulated by TGF-β and Wnt Signaling in a Smad2/3-Dvl2/3-Dependent Manner. *Plos One* 9, e85340 (2014).

3. Zhang, X. *et al.* Glycoprotein M6B Interacts with TβRI to Activate TGF-β-Smad2/3 Signaling and Promote Smooth Muscle Cell Differentiation: Glycoprotein M6B Interacts with TβRI. *Stem Cells* 37, 190–201 (2018).

4. Kordaß, T., Osen, W. & Eichmüller, S. B. Controlling the Immune Suppressor: Transcription Factors and MicroRNAs Regulating CD73/NT5E. *Front Immunol* 9, 813 (2018).

5. Chen, S. *et al.* CD73 expression on effector T cells sustained by TGF-β facilitates tumor resistance to anti-4-1BB/CD137 therapy. *Nat Commun* 10, 150 (2019).

6. Thomas, D. A. & Massagué, J. TGF-β directly targets cytotoxic T cell functions during tumor evasion of immune surveillance. *Cancer Cell* 8, 369–380 (2005).

7. Verhagen, H. J. *et al.* IGFBP7 induces apoptosis of acute myeloid leukemia cells and synergizes with chemotherapy in suppression of leukemia cell survival. *Cell Death Dis* 5, e1300–e1300 (2014).

8. Wajapeyee, N., Serra, R. W., Zhu, X., Mahalingam, M. & Green, M. R. Oncogenic BRAF Induces Senescence and Apoptosis through Pathways Mediated by the Secreted Protein IGFBP7. *Cell* 132, 363–374 (2008).

9. Koopman, G. *et al.* Annexin V for flow cytometric detection of phosphatidylserine expression on B cells undergoing apoptosis. *Blood* 84, 1415–1420 (1994).

10. Liu, J.-Z. *et al.* BDH2 triggers ROS-induced cell death and autophagy by promoting Nrf2 ubiquitination in gastric cancer. *J Exp Clin Canc Res* 39, 123 (2020).

11. Parish, I. A. *et al.* The molecular signature of CD8+ T cells undergoing deletional tolerance. *Blood* 113, 4575–4585 (2009).

12. Martinez, G. J. *et al.* The Transcription Factor NFAT Promotes Exhaustion of Activated CD8+ T Cells. *Immunity* 42, 265–278 (2015).

13. Haring, J. S. & Harty, J. T. Interleukin-18-related genes are induced during the contraction phase but do not play major roles in regulating the dynamics or function of the T-cell response to Listeria monocytogenes infection. *Infect Immun* 77, 1894–903 (2009).

14. Roychoudhuri, R. *et al.* BACH2 regulates CD8+ T cell differentiation by controlling access of AP-1 factors to enhancers. *Nat Immunol* 17, 851–860 (2016).

15. Byrne, S. M. *et al.* Cathepsin B Controls the Persistence of Memory CD8+ T Lymphocytes. *J Immunol* 189, 1133–1143 (2012).

16. Li, L. *et al.* Brief Report: Serpin Spi2A as a Novel Modulator of Hematopoietic Progenitor Cell Formation. *Stem Cells* 32, 2550–2556 (2014).

17. Gerlach, C. *et al.* The Chemokine Receptor CX3CR1 Defines Three Antigen-Experienced CD8 T Cell Subsets with Distinct Roles in Immune Surveillance and Homeostasis. *Immunity* 45, 1270–1284 (2016).

18. Förster, R., Davalos-Misslitz, A. C. & Rot, A. CCR7 and its ligands: balancing immunity and tolerance. *Nat Rev Immunol* 8, 362–371 (2008).

19. Zhang, Y., Joe, G., Hexner, E., Zhu, J. & Emerson, S. G. Host-reactive CD8+ memory stem cells in graft-versus-host disease. *Nat Med* 11, 1299–1305 (2005).

20. Gattinoni, L. *et al.* Wnt signaling arrests effector T cell differentiation and generates CD8+ memory stem cells. *Nat Med* 15, 808–813 (2009).

21. Graef, P. *et al.* Serial Transfer of Single-Cell-Derived Immunocompetence Reveals Stemness of CD8+ Central Memory T Cells. *Immunity* 41, 116–126 (2014).

22. Crompton, J. G. *et al.* Lineage relationship of CD8+ T cell subsets is revealed by progressive changes in the epigenetic landscape. *Cell Mol Immunol* 13, 502–513 (2016).

23. Gattinoni, L., Speiser, D. E., Lichterfeld, M. & Bonini, C. T memory stem cells in health and disease. *Nat Med* 23, 18–27 (2017).

24. Mariathasan, S. *et al.* TGFβ attenuates tumour response to PD-L1 blockade by contributing to exclusion of T cells. *Nature* 554, 544–548 (2018).

25. Beltra, J.-C. *et al.* Developmental Relationships of Four Exhausted CD8+ T Cell Subsets Reveals Underlying Transcriptional and Epigenetic Landscape Control Mechanisms. *Immunity* (2020) doi:10.1016/j.immuni.2020.04.014.

26. Halle, S., Halle, O. & Förster, R. Mechanisms and Dynamics of T Cell-Mediated Cytotoxicity In Vivo. *Trends Immunol* 38, 432–443 (2017).

27. Allen, F. *et al.* CCL3 augments tumor rejection and enhances CD8+ T cell infiltration through NK and CD103+ dendritic cell recruitment via IFNγ. *Oncoimmunology* 7, 00–00 (2017).

28. Bhat, P., Leggatt, G., Waterhouse, N. & Frazer, I. H. Interferon-γ derived from cytotoxic lymphocytes directly enhances their motility and cytotoxicity. *Cell Death Dis* 8, e2836–e2836 (2017).

29. Lieberman, J. The ABCs of granule-mediated cytotoxicity: new weapons in the arsenal. *Nat Rev Immunol* 3, 361–370 (2003).

30. Utzschneider, D. T. *et al.* T Cell Factor 1-Expressing Memory-like CD8+ T Cells Sustain the Immune Response to Chronic Viral Infections. *Immunity* 45, 415–427 (2016).

31. Hudson, W. H. *et al.* Proliferating Transitory T Cells with an Effector-like Transcriptional Signature Emerge from PD-1+ Stem-like CD8+ T Cells during Chronic Infection. *Immunity* 51, 1043-1058.e4 (2019).

32. Im, S. J., Konieczny, B. T., Hudson, W. H., Masopust, D. & Ahmed, R. PD-1+ stemlike CD8 T cells are resident in lymphoid tissues during persistent LCMV infection. *Proc National Acad Sci* 117, 4292–4299 (2020).

33. Siddiqui, I. *et al.* Intratumoral Tcf1+PD-1+CD8+ T Cells with Stem-like Properties Promote Tumor Control in Response to Vaccination and Checkpoint Blockade Immunotherapy. *Immunity* 50, 195-211.e10 (2019).

34. Andreatta, M. *et al.* Interpretation of T cell states from single-cell transcriptomics data using reference atlases. *Nat Commun* 12, 2965 (2021).
