# Supplementary material for: Combined PD-L1/TGFβ blockade allows expansion and differentiation of stem cell-like CD8 T cells in immune excluded tumors
Source: Nat Commun. 2023 Aug 5;14:4703. doi: 10.1038/s41467-023-40398-4 (PMC10404279; doi:10.1038/s41467-023-40398-4)
Supplement: Supplementary file 2 — Description of Additional Supplementary Files [file 41467_2023_40398_MOESM2_ESM.pdf]

Description of Additional Supplementary Files

Supplementary Data 1: T cells cluster markers

Supplementary Data 2: Whole tumor proteomic analysis

Supplementary Data 3: CD8 T cell pseudo-bulk differential expression analysis

Supplementary Data 4: CD8 T cell cluster markers (scTCR seq dataset)

Supplementary Data 5: NMF programs

Supplementary Data 6: Tumor, Myeloid cells, and Fibroblasts pseudo-bulk differential expression analysis

Supplementary Data 7: Tumor, Myeloid cells, and Fibroblasts pathway enrichment analysis
