# Supplementary material for: Combined PD-L1/TGFβ blockade allows expansion and differentiation of stem cell-like CD8 T cells in immune excluded tumors
Source: Nat Commun. 2023 Aug 5;14:4703. doi: 10.1038/s41467-023-40398-4 (PMC10404279; doi:10.1038/s41467-023-40398-4)
Supplement: Supplementary file 10 — Reporting Summary [file 41467_2023_40398_MOESM10_ESM.pdf]

nature portfolio

Corresponding author(s): Soren Muller, Shannon Turley

Last updated by author(s): July 7, 2023

Reporting Summary

Nature Portfolio wishes to improve the reproducibility of the work that we publish. This form provides structure for consistency and transparency in reporting. For further information on Nature Portfolio policies, see our Editorial Policies and the Editorial Policy Checklist.

Statistics

For all statistical analyses, confirm that the following items are present in the figure legend, table legend, main text, or Methods section.

n/a	Confirmed	
☐	☒	The exact sample size (*n*) for each experimental group/condition, given as a discrete number and unit of measurement
☒	☐	A statement on whether measurements were taken from distinct samples or whether the same sample was measured repeatedly
☐	☒	The statistical test(s) used AND whether they are one- or two-sided<br>*Only common tests should be described solely by name; describe more complex techniques in the Methods section.*
☒	☐	A description of all covariates tested
☐	☒	A description of any assumptions or corrections, such as tests of normality and adjustment for multiple comparisons
☐	☒	A full description of the statistical parameters including central tendency (e.g. means) or other basic estimates (e.g. regression coefficient) AND variation (e.g. standard deviation) or associated estimates of uncertainty (e.g. confidence intervals)
☐	☒	For null hypothesis testing, the test statistic (e.g. *F*, *t*, *r*) with confidence intervals, effect sizes, degrees of freedom and *P* value noted<br>*Give P values as exact values whenever suitable.*
☒	☐	For Bayesian analysis, information on the choice of priors and Markov chain Monte Carlo settings
☒	☐	For hierarchical and complex designs, identification of the appropriate level for tests and full reporting of outcomes
☒	☐	Estimates of effect sizes (e.g. Cohen's *d*, Pearson's *r*), indicating how they were calculated

Our web collection on statistics for biologists contains articles on many of the points above.

Software and code

Policy information about availability of computer code

Data collection	BDFACSDiva (8.0.1 and 9.1)
Data analysis	No new algorithms were developed for this manuscript. All code generated for analysis is available from the authors upon request.<br><br>Software versions:<br>Flowjo (10.8.1)<br>ImageJ/Fiji (20201104-1356)<br>Matlab (2020a)<br>Imaris (version 9.8.0)<br>GraphPad Prism (v7.02).<br>R (3.6.2, 4.1.0, 4.2.1)<br>Seurat (3.1.4)<br>Slingshot (1.4.0)<br>fgsea (1.4.0)<br>pheatmap (1.0.12)<br>CellRanger (3.1)<br>DESeq2 (1.26.0)<br>DropletUtils (1.7.16)<br>ggplot2(3.3.2)

tidyverse(1.3.1)
dplyr (1.0.6)
FSA (0.8.32)
nichenetr (1.1.1)
enrichR (3.0)
speckle (0.0.3)

For manuscripts utilizing custom algorithms or software that are central to the research but not yet described in published literature, software must be made available to editors and reviewers. We strongly encourage code deposition in a community repository (e.g. GitHub). See the Nature Portfolio guidelines for submitting code & software for further information.

Data

Policy information about availability of data

All manuscripts must include a data availability statement. This statement should provide the following information, where applicable:

- Accession codes, unique identifiers, or web links for publicly available datasets
- A description of any restrictions on data availability
- For clinical datasets or third party data, please ensure that the statement adheres to our policy

Data Availability
All unique materials used are readily available from the authors. Bulk and scRNA-seq datasets data generated in this study have been deposited in the ArrayExpress database under accession codes E-MTAB-13072 [https://www.ebi.ac.uk/biostudies/arrayexpress/studies/E-MTAB-13072] (scRNA-seq), E-MTAB-13073 [https://www.ebi.ac.uk/biostudies/arrayexpress/studies/E-MTAB-13073] (scRNA/TCR-seq), and E-MTAB-13079 [https://www.ebi.ac.uk/biostudies/arrayexpress/studies/E-MTAB-13079] (bulk RNA-seq). Proteomics data in this study have been deposited in the MassIVE database under accession code MSV000092193 [https://massive.ucsd.edu/ProteoSAFe/dataset.jsp?task=a4065d3eeb304c9f9fd5cb3ebb7b698b]. The remaining data are available within the Article, Supplementary Information or Source Data file. Source data are provided with this paper.

Code Availability
No new algorithms were developed for this manuscript. Analysis code is available through the Open Science Framework (OSF) project with ID vmb96 [https://osf.io/vmb96/?view_only=2b39e03f60a142e1bbb809847bdf5a7f].

Research involving human participants, their data, or biological material

Policy information about studies with human participants or human data. See also policy information about sex, gender (identity/presentation), and sexual orientation and race, ethnicity and racism.

Reporting on sex and gender	N/A
Reporting on race, ethnicity, or other socially relevant groupings	N/A
Population characteristics	N/A
Recruitment	N/A
Ethics oversight	N/A

Note that full information on the approval of the study protocol must also be provided in the manuscript.

Field-specific reporting

Please select the one below that is the best fit for your research. If you are not sure, read the appropriate sections before making your selection.

[x] Life sciences [ ] Behavioural & social sciences [ ] Ecological, evolutionary & environmental sciences

For a reference copy of the document with all sections, see nature.com/documents/nr-reporting-summary-flat.pdf

Life sciences study design

All studies must disclose on these points even when the disclosure is negative.

Sample size	No statistical method was used to predetermine sample size. Sample size in the mouse studies were based on preliminary experimentation and we designed our experiment to achieve a minimum n=3, but mostly n=5 (or greater) mice per group/condition. This enabled us to carry out biologically significant experiments with reproducible results.
Data exclusions	Only one animal was excluded from two analyses (figures 6C, E, F, and S7G). The animal excluded was a non-responder, the decision to exclude non-responders has been taken before data analysis and the exclusion is described in the figure legend.

Replication	The number of replicates for mouse experiments is stated in the figure legends. The in vitro experiment shown in supplementary figure 7H was performed once with three technical replicates per condition. All other experiments/figures are based on public data from patients and were not replicated in our own lab given that we don't have access to these or similar tissues.
Randomization	Mice were grouped using the "Gould-Hanson with Chan modification Grouping Algorithm" as follows: (Step 1) Determine average volume and remove any ungrouped animals based on their deviation from the average; (Step 2) Sort list of animals by alternating descending deviant high and low volumes (H3,L3,H2,L2,H1,L1); (Step 3) Assign to groups based on even or odd number of groups (to create even distribution): (i) Odd # of groups, in straight sequential (1,2,3,4,5,1,2,3,4,5...etc) and (ii) Even # of groups, in snaking-sequential (1,2,3,4,4,3,2,1...etc); (Step 4) All groups are assigned sequentially with the other groups until all animals in that group are assigned, i.e. larger groups will have the least variation in the extra animals). For non animal experiments, randomization was not required as studies in human subjects were focused on interferon response across all samples and patients as a whole.<br><br>For the in vitro experiment shown in supplementary figure 7H cells were distributed evenly in a 96 well plate and the assignment to a group of treatment was determined by the position on the plate.
Blinding	In all animal studies, blinding was not necessary. Animal were grouped according to genotype/treatment.

Reporting for specific materials, systems and methods

We require information from authors about some types of materials, experimental systems and methods used in many studies. Here, indicate whether each material, system or method listed is relevant to your study. If you are not sure if a list item applies to your research, read the appropriate section before selecting a response.

Materials & experimental systems

n/a	Involved in the study
☐	☒ Antibodies
☐	☒ Eukaryotic cell lines
☒	☐ Palaeontology and archaeology
☐	☒ Animals and other organisms
☐	☒ Clinical data
☒	☐ Dual use research of concern
☒	☐ Plants

Methods

n/a	Involved in the study
☒	☐ ChIP-seq
☐	☒ Flow cytometry
☒	☐ MRI-based neuroimaging

Antibodies

Antibodies used	All the antibodies are listed in a Supplementary Table included within the Supplementary Information file. All the dilutions used are included in the method section as concentration used for the staining when the manufactured listed the stock concentration in the antibody data sheet or as dilution used when that information was not available.
Validation	The anti-mouse programmed death-ligand 1 (PD-L1 IgG1 monoclonal antibody (Clone 6E11) functional blocking pre-clinical was generated at Genentech by immunization of PD-L1 knockout mice with a PD-L1-Fc fusion protein and cloned onto a murine IgG1 isotype (doi:10.1038/s43018-020-0075-x).<br><br>The anti-gp120 mIgG1 antibody used as isotype control for in vivo studies were generated and validated in vivo and in vitro by Genentech (this antibody is the same used in doi: 10.1038/nature25501).<br><br>The anti-TGFb mIgG1 antibodies used for in vivo studies were generated by Genentech. The anti-TGFb antibodies ability to bind and block all three isoforms of TGFb were assessed in vitro and in vivo by potency and binding assays. For the experiments included in this paper we used two anti-TGFb clones generated in house that differ by three amino acids and have similar properties in blocking TGFb: 1D11 (Affinity (pM): TGFb1 = 52 ± 1, TGFb2 = 201 ± 93, TGFb3 = 62 ± 1; Potency IC50 (nM): TGFb1 = 1.4, TGB2 = 12, TGFb3 = 0.13; this antibody is the same used in doi: 10.1038/nature25501) used in 6 out of 17 in vivo experiments, and 1D11.v8.13.EG (Affinity (pM): TGFb1 = 19 ± 2, TGFb2 = 283 ± 30, TGFb3 = 20 ± 3; Potency IC50 (nM): TGFb1 = 0.72, TGB2 = 2.2, TGFb3 = 0.026; this antibody is the same described in doi: 10.1093/toxsci/kfaa024) used in 11 out of 17 experiments.<br><br>The anti-IFNg neutralizing antibody was obtained from BioXcell and has been validated by several groups in published papers (https://bioxcell.com/invivomab-anti-mouse-ifng-be0055#tab_references).<br><br>The anti-CD8a antibody used for IHC (clone 1.21E3.1.3) is a Genentech-proprietary mixed hamster IgG. Validation was performed on mCD8-transfected and non-transfected HEK293 cell pellets that were formalin-fixed and paraffin-embedded as positive and negative controls, respectively. Sections of mouse spleen and thymus as well as a normal mouse tissue array containing kidney, liver, pancreas, stomach, spleen, large intestine, small intestine, lung, skeletal muscle, heart, lymph node, esophagus, thyroid, trachea, ovary, uterus, bladder, eye, bone marrow, brain (cerebellum, cerebral cortex, hippocampus), adrenal gland, mammary gland and skin were also included. CD8a antibody was evaluated for sensitivity and specificity on all samples over a range of concentrations (1, 2.5, 5 and 10 ug/mL) by a pathologist and a working concentration (5 ug/mL) that optimized sensitivity and signal to noise was selected based on staining in tissues.<br><br>For all the other antibodies we reported the clone, the catalogue number and the manufacturer information in the Supplementary

Table included within the Supplementary Information file: all the validations and references can be found in the manufacture's websites.

Eukaryotic cell lines

Policy information about cell lines and Sex and Gender in Research

Cell line source(s)	EMT6: Original source ATCC and then screened and stored by common cell repository at Genentech. EMT6 cells were engineered to express either THY1.1 (for flow cytometry and RNAseq experiments), mAPPLE (for in vivo imaging) or for IFNGR1 deletion. Generation of these cell lines is described in the method section.<br><br>HEK293T: Original source ATCC and then stored by common cell repository at Genentech.
Authentication	EMT6 cells were analyzed by RNAseq.
Mycoplasma contamination	Cell lines are routinely screened and the cells used in studies described in this manuscript were negative for mycoplasma.
Commonly misidentified lines<br>(See ICLAC register)	No commonly misidentified cell lines were used in the study

Animals and other research organisms

Policy information about studies involving animals; ARRIVE guidelines recommended for reporting animal research, and Sex and Gender in Research

Laboratory animals	As stated in the Methods, 8-10 week old female Balb/c mice from Charles River Laboratories (Hollister, CA). The IFNg-YFP reporter mice were licensed from UCSF and backcrossed to the BALB/c background. All mice were housed at Genentech in individually ventilated cages within animal rooms maintained on a 14:10-hour, light:dark cycle. Animal rooms were temperature and humidity-controlled, between 68-79°F and 30-70% respectively, with 10 to 15 room air exchanges per hour. Mice were acclimated to study conditions for at least 3 days before tumor cell implantation. Animals were 8-10 weeks old. Only animals that appeared to be healthy and free of obvious abnormalities were used for the studies.
Wild animals	No wild animals were used in the study.
Reporting on sex	Only female mice were used. This information is reported in the method section.
Field-collected samples	No field collected samples were used in the study.
Ethics oversight	All animal studies were approved by the Genentech Institutional Animal Care and Use Committee.

Note that full information on the approval of the study protocol must also be provided in the manuscript.

Clinical data

Policy information about clinical studies

All manuscripts should comply with the ICMJEguidelines for publication of clinical research and a completedCONSORT checklist must be included with all submissions.

Clinical trial registration	*Provide the trial registration number from ClinicalTrials.gov or an equivalent agency.*
Study protocol	*Note where the full trial protocol can be accessed OR if not available, explain why.*
Data collection	*Describe the settings and locales of data collection, noting the time periods of recruitment and data collection.*
Outcomes	*Describe how you pre-defined primary and secondary outcome measures and how you assessed these measures.*

Flow Cytometry

Plots

Confirm that:

- [x] The axis labels state the marker and fluorochrome used (e.g. CD4-FITC).
- [x] The axis scales are clearly visible. Include numbers along axes only for bottom left plot of group (a 'group' is an analysis of identical markers).
- [x] All plots are contour plots with outliers or pseudocolor plots.
- [x] A numerical value for number of cells or percentage (with statistics) is provided.

Methodology

Sample preparation	Sample preparation for each flow cytometry experiment is detailed in the methods section. Briefly, before any staining procedure tumors were weighed and enzymatically digested using a cocktail of dispase, collagenase P and DNaseI for 45 min at 37°C, to obtain a single-cell suspension. Cells were counted using a Vi-CELL XR (Beckman Coulter, Brea, CA).
Instrument	Cells were sorted using a BD FACSAria™ Fusion flow cytometer (BD Biosciences, San Jose, CA). Flow Cytometry data were collected with a BD LSRFortessa cell analyzer or FACSymphony (BD Biosciences, San Jose, CA).
Software	Flow data were analyzed in FlowJo, exported to csv files and statistical analysis was performed using R package tidyverse (version 1.3.1), dplyr (version 1.0.6), and FSA (version 0.8.32).
Cell population abundance	For bulk and scRNAseq, four population were sorted: T cell (7AAD- Calcein blue+ CD45+ THY1.1- TCRb+), fibroblasts (7AAD- Calcein Blue+ CD45- THY1.1- CD31- PDPN+), myeloid cells (7AAD- Calcein Blue+ CD45+ THY1.1- CD11b+) and tumor cells (7AAD- Calcein Blue+ CD45- THY1.1+). For scRNA-seq, 50k cells for each population were sorted in 300 UL of MACS buffer kept at 4C. Cells were then counted and resuspended at an adequate concentration for loading into the 10X chips. For bulk RNA-seq, 5k cells for each population per mouse were sorted in 100 UL of RA1 buffer + 2 UL of TCEP (NucleoSpin RNA XS) spun down and immediately frozen in dry ice. Samples were conserved at -80C until RNA extraction. For TCR/scRNA-seq,T cells form the tumor were sorted as 7AAD-CB+ CD45+ CD11b-CD19-NK1.1-CD90+. 75k cells T cells were sorted in 300 UL of MACS buffer kept at 4C. Cells were then counted and resuspended at an adequate concentration for loading into the 10X chip. For bulk and scRNAseq, four population were sorted: T cell (7AAD- Calcein blue+ CD45+ THY1.1- TCRb+), fibroblasts (7AAD- Calcein Blue+ CD45- THY1.1- CD31- PDPN+), myeloid cells (7AAD- Calcein Blue+ CD45+ THY1.1- CD11b+) and tumor cells (7AAD- Calcein Blue+ CD45- THY1.1+). For scRNA-seq, 50k cells for each population were sorted in 300 UL of MACS buffer kept at 4C. Cells were then counted and resuspended at an adequate concentration for loading into the 10X chips. For bulk RNA-seq, 5k cells for each population per mouse were sorted in 100 UL of RA1 buffer + 2 UL of TCEP (NucleoSpin RNA XS) spun down and immediately frozen in dry ice. Samples were conserved at -80C until RNA extraction. For TCR/scRNA-seq,T cells form the tumor were sorted as 7AAD-CB+ CD45+ CD11b-CD19-NK1.1-CD90+. 75k cells T cells were sorted in 300 UL of MACS buffer kept at 4C. Cells were then counted and resuspended at an adequate concentration for loading into the 10X chip. For Antigen specific TCR/scRNA-seq, E22 tet+ CD8 T cells form the tumor were sorted as 7AAD-CB+ CD45+ CD11b-CD19-NK1.1-CD90+CD8+ E22+. 16 to 50k cells T cells were sorted in 300 UL of MACS buffer kept at 4C. Cells were then counted and resuspended at an adequate concentration for loading into the 10X chip.
Gating strategy	For bulk and scRNAseq, four population were sorted: T cell (7AAD- Calcein blue+ CD45+ THY1.1- TCRb+), fibroblasts (7AAD- Calcein Blue+ CD45- THY1.1- CD31- PDPN+), myeloid cells (7AAD- Calcein Blue+ CD45+ THY1.1- CD11b+) and tumor cells (7AAD- Calcein Blue+ CD45- THY1.1+). For TCR/scRNA-seq, T cells form the tumor were sorted as 7AAD-CB+ CD45+ CD11b-CD19-NK1.1-CD90+. For Antigen specific TCR/scRNA-seq, E22 tet+ CD8 T cells form the tumor were sorted as 7AAD-CB+ CD45 + CD11b-CD19-NK1.1-CD90+CD8+ E22+.

☒ Tick this box to confirm that a figure exemplifying the gating strategy is provided in the Supplementary Information.
